# Rapid and comprehensive diagnostic method for repeat expansion diseases using nanopore sequencing

**DOI:** 10.1038/s41525-022-00331-y

**Published:** 2022-10-26

**Authors:** Satoko Miyatake, Eriko Koshimizu, Atsushi Fujita, Hiroshi Doi, Masaki Okubo, Taishi Wada, Kohei Hamanaka, Naohisa Ueda, Hitaru Kishida, Gaku Minase, Atsuhiro Matsuno, Minori Kodaira, Katsuhisa Ogata, Rumiko Kato, Atsuhiko Sugiyama, Ayako Sasaki, Takabumi Miyama, Mai Satoh, Yuri Uchiyama, Naomi Tsuchida, Haruka Hamanoue, Kazuharu Misawa, Kiyoshi Hayasaka, Yoshiki Sekijima, Hiroaki Adachi, Kunihiro Yoshida, Fumiaki Tanaka, Takeshi Mizuguchi, Naomichi Matsumoto

**Affiliations:** 1 Department of Human Genetics, Yokohama City University Graduate School of Medicine, Yokohama, Kanagawa 236-0004 Japan; 2 Department of Clinical Genetics, Yokohama City University Hospital, Yokohama, Kanagawa 236-0004 Japan; 3 Department of Neurology and Stroke Medicine, Yokohama City University Graduate School of Medicine, Yokohama, Kanagawa 236-0004 Japan; 4 Department of Neurology, Yokohama City University Medical Center, Yokohama, Kanagawa 232-0024 Japan; 5 Department of Obstetrics and Gynecology, Asahikawa Medical University, Asahikawa, Hokkaido 078-8510 Japan; 6 Department of Medicine (Neurology and Rheumatology), Shinshu University School of Medicine, Matsumoto, Nagano 390-8621 Japan; 7 Department of Neurology, National Hospital Organization Higashisaitama National Hospital, Hasuda, Saitama 349-0196 Japan; 8 Department of Pediatrics, National Hospital Organization Higashisaitama National Hospital, Hasuda, Saitama 349-0196 Japan; 9 Department of Neurology, Graduate School of Medicine, Chiba University, Chiba, 260-8677 Japan; 10 Department of Pediatrics, Yamagata University School of Medicine, Yamagata, Yamagata 990-9585 Japan; 11 Department of Rare Disease Genomics, Yokohama City University Hospital, Yokohama, Kanagawa 236-0004 Japan; 12 Riken Center for Advanced Intelligence Project, 1-4-1 Nihonbashi, Chuo-ku, Tokyo 103-0027 Japan; 13 Department of Pediatrics, Miyukikai Hospital, Social Medical Corporation Miyuki, Kaminoyama, Yamagata 999-3161 Japan; 14 Department of Neurology, University of Occupational and Environmental Health School of Medicine, Kitakyushu, Fukuoka 807-8555 Japan; 15 Department of Neurology, JA Nagano Kouseiren, Kakeyu-Misayama Rehabilitation Center, Kakeyu Hospital, Kakeyuonsen 1308, Ueda, 386-0396 Japan

**Keywords:** Genetic testing, Genetics research

## Abstract

We developed a diagnostic method for repeat expansion diseases using a long-read sequencer to improve currently available, low throughput diagnostic methods. We employed the real-time target enrichment system of the nanopore GridION sequencer using the adaptive sampling option, in which software-based target assignment is available without prior sample enrichment, and built an analysis pipeline that prioritized the disease-causing loci. Twenty-two patients with various neurological and neuromuscular diseases, including 12 with genetically diagnosed repeat expansion diseases and 10 manifesting cerebellar ataxia, but without genetic diagnosis, were analyzed. We first sequenced the 12 molecularly diagnosed patients and accurately confirmed expanded repeats in all with uniform depth of coverage across the loci. Next, we applied our method and a conventional method to 10 molecularly undiagnosed patients. Our method corrected inaccurate diagnoses of two patients by the conventional method. Our method is superior to conventional diagnostic methods in terms of speed, accuracy, and comprehensiveness.

## Introduction

Tandem repeats (TRs) are a common form of genetic variation in the human genome^1^. Their expansion may cause disease, usually manifesting a neurological phenotype. To date, approximately 60 TR loci are associated with more than 69 diseases when expanded^2^. Repeat expansion is responsible for the most common genetic neurology conditions^3^, whose devastating clinical courses urgently require effective neurotherapies. Very recently, promising neurotherapeutic approaches, such as antisense oligonucleotides^4,5^, small compounds^6^, and antibodies^7^, have been reported for various neurodegenerative diseases. For all these approaches, accurate molecular diagnosis is required.

Molecular diagnosis of repeat expansion disease has been a challenge for neurologists. First, locus heterogeneity is common, necessitating multiple experiments to examine possible loci. Second, TRs are often GC-rich and elongated, making polymerase chain reaction (PCR) difficult. Conventional diagnostic methods are mainly PCR-based, such as flanking PCR and fragment analysis of expanded regions, or repeat-primed PCR (RP-PCR), or depend on southern blotting; therefore, PCR conditions/primers or probes must be specifically set up for each locus. However, this is time consuming and technically difficult, requiring extensive optimization, which can sometimes not be achieved. In practice, several disease loci, but not all, are chosen for diagnosis, sometimes resulting in incomplete screening.

Short-read next-generation sequencing has only contributed to finding new disease-causing repeat expansions to a limited extent; however, long-read sequencing using platforms from Oxford Nanopore Technologies or Pacific Biosciences can potentially cover entire repeat expansions and overcome low-complexity and GC-rich genomic regions. They have boosted recent repeat expansion disease discoveries involving difficult sequences, such as GC-rich repeat motifs^8,9^ or repeat motifs that are different from those in the reference genome^1,10–13^. The currently available methods to capture a region of interest, such as PCR-based enrichment, Cas9-mediated PCR-free enrichment^14,15^ or Read Until^16–18^, target enrichment software that does not require prior sample preparation, enable long-read sequencing of a targeted region, and they have been recently applied to human genetic research^19,20^.

Here, we developed a diagnostic method for repeat expansion diseases using long-read sequencing in a clinical setting. We employed real-time target enrichment using a nanopore GridION sequencer with the adaptive sampling option, which is an implemented version of Read Until on GridION, whereby software-based assignment of target regions is available for any repeat expansion disease-associated locus of interest, for up to 1% of a whole-genome region. This enables the GridION sequencer to selectively sequence only the DNA fragments of targets. Furthermore, we built an analysis pipeline that prioritized the disease-causing loci using tandem-genotypes^21^, a bioinformatic tool that finds changes in the length of TRs from “long” DNA reads aligned to a genome. The pipeline then generates a list of loci with large repeat copy number changes in patients compared with control data in the order of pathogenic possibility. We could easily detect pathogenic repeat expansion without specialist expertise in repeat expansion diseases by following the prioritized list and our data evaluation flowchart. In addition, detailed repeat analyses, such as consensus sequence generation and characterization of interrupting sequence, or methylation analysis, could be performed when needed. From our pilot study of 22 patients with neurological and neuromuscular disorders, we confirm that our diagnostic method is faster, more accurate, and more comprehensive compared with conventional methods.

## Results

### Evaluation of sequencing quality

**Fig. 1:**
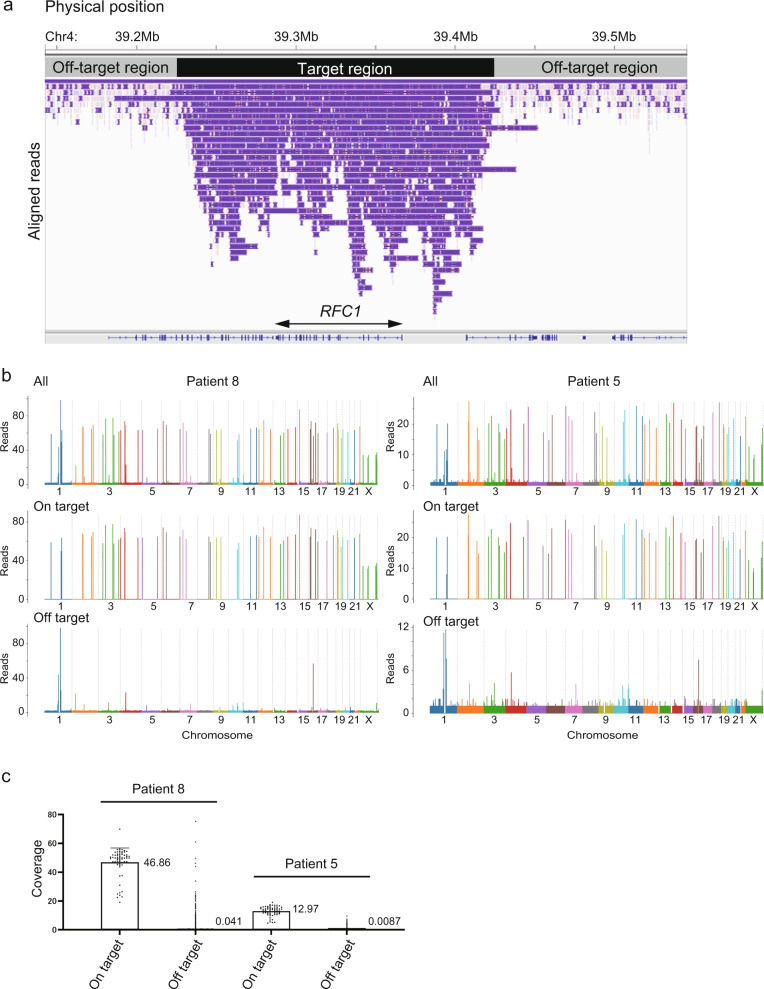
Successful capture of targeted regions with relatively homogeneous coverage. **a** The integrative genomics viewer (IGV) depicting the entire *RFC1* region successfully captured by adaptive sampling, as an example of successful target enrichment in Patient 9. **b** Coverage plots for Patient 8 (left) and Patient 5 (right). Upper, middle, and lower panels show coverage plots across whole chromosomes of all reads, on-target reads, and off-target reads, respectively. **c** All per-locus coverage of “on-target” and coverage per every 5000 bp of “off-target” reads by adaptive sampling were plotted for Patients 8 and 5. The average coverage depths for on- and off-targets are presented in the graph.

Fifty-nine targeted loci were successfully captured with a mean coverage depth of 24.7 for all 22 patients. Figure 1a shows an example of successful capture at the *RFC1* locus. Median depth of coverage across all 59 targeted loci was generally homogenous except for *NOTCH2NLC*, which might be due to its location in a segmentally duplicated region or its paralogous genes, such as *NOTCH2NLA, NOTCH2NLB*, and *NOTCH2NLR* (Supplementary Fig. 1). For detailed evaluation, we plotted the coverage depth of a single run on respective chromosomes for two patients: Patient 8 who had relatively high depth and Patient 5 who had less depth. Relatively homogenous coverage was replicated for on-target reads in these samples. Off-target reads were generally scarce across all chromosomes, although several off-target loci were commonly observed in two patients with relatively high coverage depth (Fig. 1b). Manual inspection revealed that most of these off-target regions did not encompass coding genes, but were located within repetitive regions or at the centromere (data not shown). Even with several highly covered off-targets, targeted regions seemed very accurately enriched overall because the average per-locus coverage depth for on-targets (46.86× for Patient 8 and 12.97× for Patient 5) was roughly 1000 times larger than that for off-targets (0.041× for Patient 8 and 0.0087× for Patient 5) among all selected reads (Fig. 1c).

### Repeat expansions precisely identified in the validation study

**Fig. 2:**
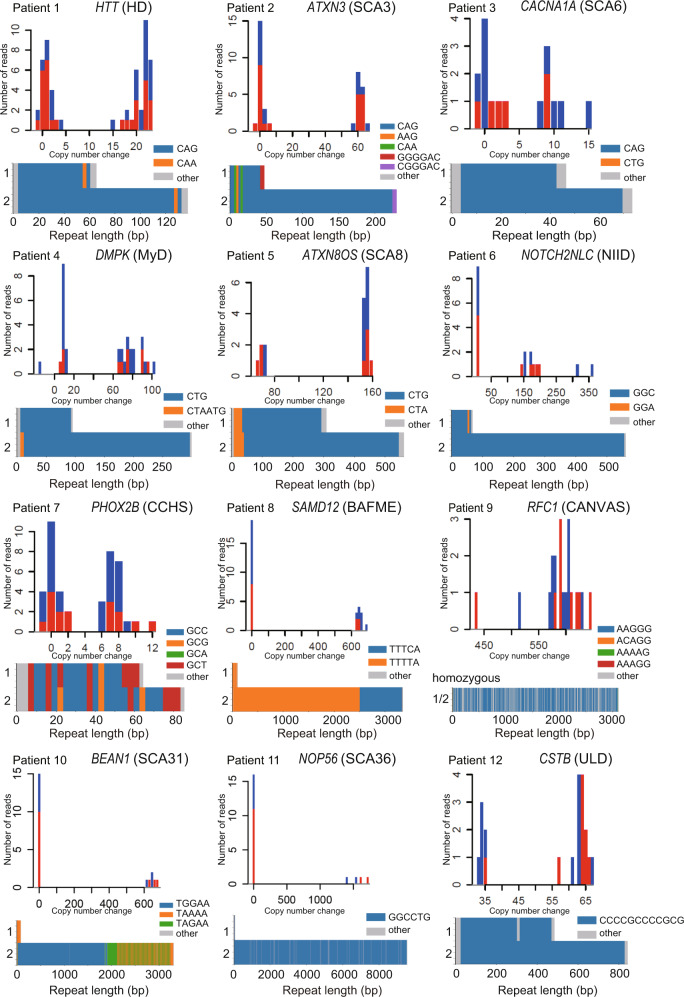
Pathogenic repeat expansions identified in all positive control samples. Histograms of tandem-genotypes output and waterfall plots for all positive controls in the validation study are shown. For the histograms, the X-axis indicates copy number change compared with the number of repeat units in the reference human genome: 21 for *HTT*, 10 for *ATXN3*, 13 for *CACNA1A*, 20 for *DMPK*, 15 for *ATXN8OS*, 13 for *NOTCH2NLC*, 20 for *PHOX2B*, 20 for *SAMD12*, 11 for *RFC1*, 13 for *BEAN1*, 4 for *NOP56*, and 3 for *CSTB*. The waterfall plot was generated using either hac mode (Patients 1, 2, 3, 7, 8, 9) or sup mode (Patients 4, 5, 6, 10, 11, 12).

**Fig. 3:**
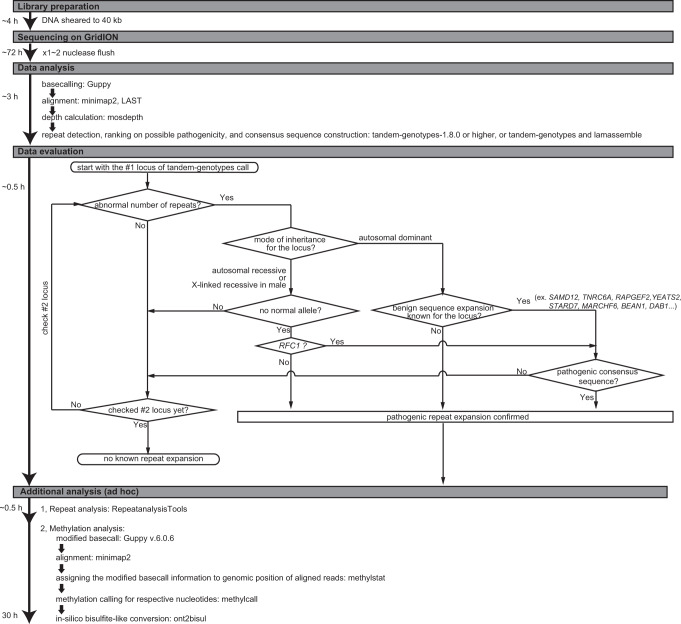
Diagnostic flowchart for human repeat expansion diseases using the GridION sequencer. The flowchart for library preparation, sequencing, data analysis, data evaluation, and additional analysis (in an ad hoc manner) is shown along with the estimated time required for each step.

All pathogenic repeat expansions irrespective of repeat unit sequence and length were detected in 12 positive controls (Patients 1–12, Table 1, and Fig. 2) and were ranked first in 10 and second in 2 patients by our prioritization workflow (Fig. 3). For validation and discovery studies, loci ranked for possible pathogenicity in each patient are presented in Supplementary Tables 1 and 2, respectively. For patients whose pathogenic locus was ranked second, polymorphic *TNRC6A* repeat expansion in Patient 3 and heterozygous *RFC1* repeat expansion in Patient 4 were ranked as #1. For Patient 3, a *TNRC6A* repeat expansion was judged as a polymorphism by examining the consensus sequence constructed from our workflow (Supplementary Fig. 2). For Patient 4, an *RFC1* repeat expansion was heterozygous; therefore, this patient was considered a carrier by calculating the number of repeat units in the two respective alleles using tandem-genotypes (Supplementary Table 1).

**Table 1:** Comparison of conventional methods with targeted nanopore sequencing and adaptive sampling.

			Conventional method			Target-enriched long-read sequencing							
Patient	Clinical diagnosis	Previous genetic diagnosis	Result	Mean depth across all loci (×)	Number of reads spanning expanded repeat (×)	Result	Match with conventional test	Rank	Gene	Repeat unit sequence	Number of repeat units	SCA31-linked SNP	Basecalling in sup mode
*Validation study*													
Patient 1		HD	Number of repeat units: 22/46 (20/44 for CAG repeat only)	43.3	31	Detected	–	1	*HTT*	CAG	18/42		
Patient 2		SCA3	Abnormally large flanking PCR amplicon	22.4	16	Detected	–	1	*ATXN3*	CAG	11/72		
Patient 3		SCA6	Number of repeat units: 13/22	18.0	7	Detected	–	2	*CACNA1A*	CAG	13/22		
Patient 4		MyD	Number of repeat units: approximately 100	22.8	18	Detected	–	2	*DMPK*	CTG	29/95		+
Patient 5		SCA8	Abnormally large flanking PCR amplicon	13.0	17	Detected	–	1	*ATXN8OS/ATXN8*	CTG·CAG	86/168 (CTA = 9/11)		+
Patient 6		NIID	Positive RP-PCR	25.0	10	Detected	–	1	*NOTCH2NLC*	GGC	19/185		+
Patient 7		CCHS	27 polyalanine repeat expansion	39.0	21	Detected	–	1	*PHOX2B*	GCX (X=C/G/A/T)	19/26		
Patient 8^a^		BAFME	Positive PR-PCR, Expanded repeat length: approximately 3500 bp	47.4	11	Detected	–	1	*SAMD12*	TTTCA	0/167 (TTTTA = 17/500)		
Patient 9^a^		CANVAS	Positive RP-PCR, Expanded repeat length: approximately 3527 bp	29.1	20	Detected	–	1	*RFC1*	AAGGG	622/622		
Patient 10		SCA31	Abnormally large flanking PCR amplicon, Positive RP-PCR. Positive SCA31-linked SNP	24.7	7	Detected	–	1	*BEAN1*	TGGAA	0/373	+	+
Patient 11		SCA36	Positive RP-PCR	28.1	4	Detected	–	1	*NOP56*	GGCCTG	10/1593		+
Patient 12^a^		ULD	Expanded repeat length: approximately 400/800 bp with abnormal repeat unit sequence	26.1	20	Detected	–	1	*CSTB*	CCCCGCCCCGCG	40/70		+
Individual 1^a^	None	Carrier	Heterozygous repeat expansion with varied length and hypermethylation in *NOTCH2NLC*	23.5	7	Detected		1	*NOTCH2NLC*	GGC	26/510m		
*Discovery study*													
Patient 13	SCA	None	1st result: no pathogenic repeat expansion, Revised result: SCA6 (Number of repeat units: 17/21)	34.0	14	SCA6	No	1	*CACNA1A*	CAG	16/21		
Patient 14	SCA	None	SCA31 (abnormally large flanking PCR amplicon, positive PR-PCR, negative SCA31-linked SNP)	15.2	3	SCA31	Yes	1	*BEAN1*	TGGAA	0/249	–	+
Patient 15	SCA	None	SCA6 (number of repeat units: 21/22)	23.1	13	SCA6	Yes	1	*CACNA1A*	CAG	20/21		
Patient 16	SCA	None	SCA31 (abnormally large flanking PCR amplicon, positive PR-PCR, positive SCA31-linked SNP)	26.7	8	SCA31	Yes	1	*BEAN1*	TGGAA	0/269	+	+
Patient 17	SCA	None	1st result: SCA8, revised result: intermediate expansion in SCA8 locus	15.5	NA	Intermediate expansion in SCA8 locus	No	1	*ATXN8OS/ATXN8*	CTG·CAG	12/47 (CTA = 8/19)		
Patient 18	CANVAS	None	CANVAS (positive RP-PCR, expanded repeat length: 4199/5140 bp)	19.6	10	CANVAS	Yes	1	*RFC1*	AAGGG	714/988		
Patient 19	CANVAS	None	CANVAS (positive RP-PCR, expanded repeat length: 4346 bp)	19.8	9	CANVAS	Yes	1	*RFC1*	AAGGG	682/927		
Patient 20	SCA	None	No known repeat expansion	17.1	NA	Undetected	Yes	–	–	–	–		
Patient 21	SCA	None	No known repeat expansion	18.7	NA	Undetected	Yes	–	–	–	–		
Patient 22	SCA	None	No known repeat expansion	15.2	NA	Undetected	Yes	–	–	–	–		

For the number of repeat units, we counted how many repeat units appeared in the consensus long-read sequence except for *RFC1*, *NOP56*, and *CSTB*-associated repeat expansion with frequent contaminating other sequences, in which we calculated the number of repeat units as the total expanded repeat length divided by the nucleotide count of the authentic repeat unit. For Patients 5, 8, and 17, the number of benign repeat units is shown in brackets. For Individual 1, “m” following the number of repeat units means methylated repeat expansion. For the fragment analysis performed on Patient 1, the number of repeat units might be 1–3 more than it really was because the PCR amplified the CCG repeat as well as the CAG repeat. Because target-enriched long-read sequencing on GridION identified that this patient had 11 CCG repeats on both alleles (two more than the reference), the number of repeat units for CAG was estimated at 20/44.

*BAFME* benign adult familial myoclonic epilepsy, *CANVAS* cerebellar ataxia, neuropathy, vestibular areflexia syndrome, *CCHS* congenital central hypoventilation syndrome, *HD* Huntington’s disease, *MyD* myotonic dystrophy, *NIID* neuronal intranuclear inclusion disease, *RP-PCR* repeat-primed PCR, *SCA* spinocerebellar ataxia, *ULD* Unverricht–Lundborg disease.

^a^Patients 8^45^, 12^45^, 9^34^, and Individual 1^24^ were previously described.

Single targeted long-read sequencing (T-LRS) analysis provided comprehensive results, including whole repeat sequence and expanded repeat length/number of repeat units with its distribution, while conventional methods only provided some of this information for one repeat locus, such as specific repeat unit sequence (RP-PCR), expanded repeat length (southern blotting) or the number of repeat units (fragment length analysis), or only implied that the locus was disease-causing without any detailed data (flanking PCR). Furthermore, the nucleotide-level resolution of T-LRS provided precise information on any interrupting sequences near disease-causing repeats, which can act as disease modifiers or markers. In Patient 1, expanded CAG repeats ended with (CAACAG)_1_, indicating that this patient will follow the average disease course and severity. Gain or loss of CAACAG sequence influences the age of onset and severity^22^, which can be a prognostic marker. In Patient 2, CGG, which is associated with intergenerational instability, was confirmed in the mutant allele. In Patient 4, CTA-ATG sequence was inserted at the 5′ end of an expanded repeat. This sequence remained after sequences were basecalled again with super accuracy (sup) mode. Various interrupting sequences, such as CCG, CGG, CAG, and CTC, in the *DMPK* repeat expansion at the 3′ end and more rarely at the 5′ end have been reported with an estimated frequency of 3–5%, and were associated with a milder phenotype^23^. The CTA-ATG sequence found in this patient is a previously unreported interrupting sequence with undetermined clinical significance, although we could not exclude the possibility of sequencing error. In Patient 5, pathogenic CTG expansions were observed in both alleles, indicating that this patient had biallelic expansion. The waterfall plot of this patient showed the sequence content of the entire repeat, including a benign CTA repeat, and a disease-causing CTG repeat. The entire repeat sequence content is difficult to clarify using conventional methods, and its pathogenicity was determined based on the total repeat length, which did not exclude the large benign CTA repeat expansion from the pathogenic one. Our T-LRS overcomes this difficulty. In Patient 6, the normal allele had a GGA interruption, which may reduce GGC repeat instability^24^. In Patient 7, the waterfall plot clearly showed not only repeat length abnormality, but also the detailed sequence content of GCX, where X is A/T/G/C. In Patient 9, homozygous AAGGG repeat expansion was detected. In Patient 10, pathogenic TGGAA, polymorphic TAAAA (common to all ethnic groups), and TAGAA (common in Japanese) repeat unit sequences were all confirmed (Fig. 2).

For Patients 4 and 6 and Individual 1, methylation analysis was performed. Patient 4 was diagnosed with an adult-onset, mild form of myotonic dystrophy having relatively short repeat expansion (approximately 100 repeats). Based on the recent paper reporting that abnormal methylation is mostly observed in the congenital form of myotonic dystrophy, and that patients with larger expanded alleles are more likely to show abnormal methylation^25^, expanded repeat in this patient may not be hypermethylated. Patient 6 is diagnosed with neuronal intranuclear inclusion disease (NIID); therefore the expanded repeat is expected to be unmethylated. Individual 1 is an unaffected father with an extremely long and hypermethylated repeat expansion in *NOTCH2NLC*^24^. As expected, the pathogenic repeat expansions in Patients 4 and 6 were not methylated, while an extremely long repeat expansion in asymptomatic Individual 1 was hypermethylated (Supplementary Fig. 3).

### Repeat expansions identified in previously undiagnosed patients

**Fig. 4:**
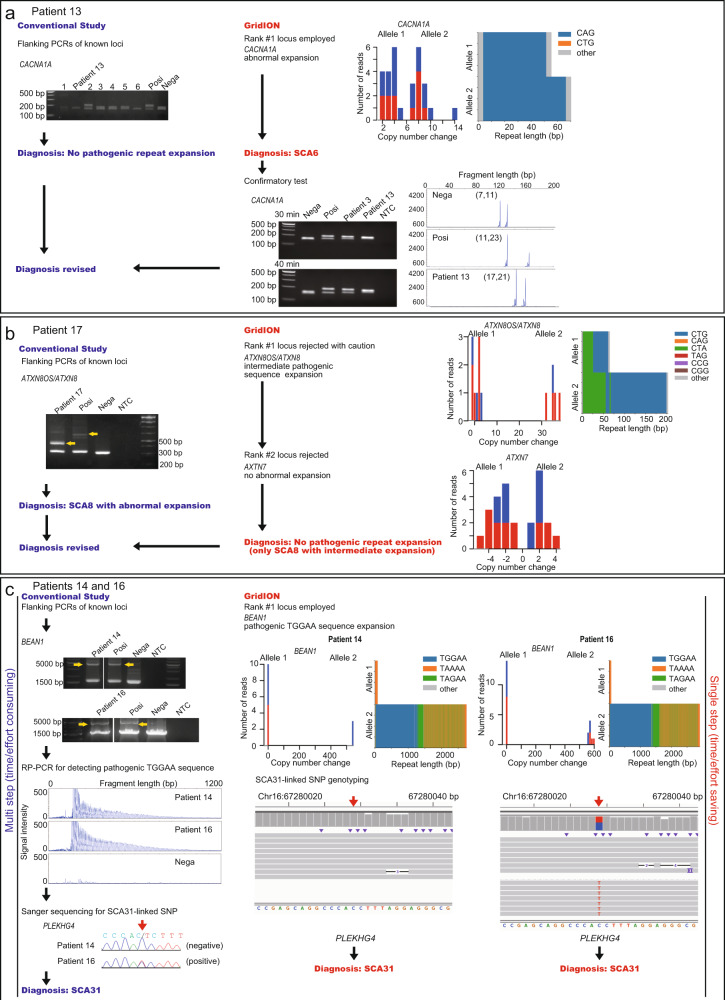
Examples of four samples for three loci in the discovery study. For **a**–**c**, the conventional study results are shown on the left and the results of our diagnostic method using GridION are shown on the right. Posi positive control, Nega negative control, NTC no template control, RP-PCR repeat-primed PCR. **a** Upper left panel shows the flanking PCR result for the *CACNA1A* locus. Patient 13 and six other patients (1–6) were tested using a 2% agarose gel and only the patient labeled “2” was judged as positive. Upper right panel shows T-LRS results detecting *CACNA1A* as the rank #1 locus. Lower right panel shows the confirmatory flanking PCR and fragment analysis of the *CACNA1A* locus. Flanking PCR was evaluated on a 2.5% agarose gel. **b** Left panel shows flanking PCRs result for the *ATXN8OS/ATXN8* locus. Right panel shows the T-LRS result detecting *ATXN8OS/ATXN8* as the rank #1 locus but with ambiguous pathogenicity. The rank #2 locus was also rejected for pathogenicity. **c** Left panel shows an abnormally expanded PCR amplicon in *BEAN1*, RP-PCR detecting a pathogenic TGGAA repeat, and Sanger sequencing for an SCA31-linked SNP in which one of the patients (Patient 14) did not have this SNP. Right panel shows the T-LRS result detecting the rank #1 locus as *BEAN1* and SCA31-linked SNP genotyping shown in the integrative genomics viewer. The result was matched between the two methods, but multiple experiments were needed with the conventional method.

We tested whether we could detect disease-causing repeat expansions in molecularly undiagnosed patients using our method. We examined 10 such patients who had been clinically diagnosed with spinocerebellar ataxia (SCA) (*n* = 8) or cerebellar ataxia, neuropathy, vestibular areflexia syndrome (CANVAS) (*n* = 2). Two groups of researchers, who were blind to the results of the respective methods, were assigned to analyze all 10 patients by conventional methods or T-LRS. Both the conventional methods and T-LRS diagnosed 6 out of the 10 patients. Results were different between the conventional methods and T-LRS in two patients (Patients 13 and 17 as described below). For both, the conventional method diagnosis was revised and the T-LRS results were found to be correct (Table 1 and Fig. 4). The results were matched in the remaining patients.

### Case studies

#### Patient 13

CAG expansion in *CACNA1A*, ranging from 20 to 32 repeats, causes SCA6^26^. The normal repeat unit limit of 18 is close to the abnormal threshold of 20 repeat units; therefore, it may be difficult to diagnose a patient with SCA6 by a conventional method, such as flanking PCR.

**Fig. 5:**
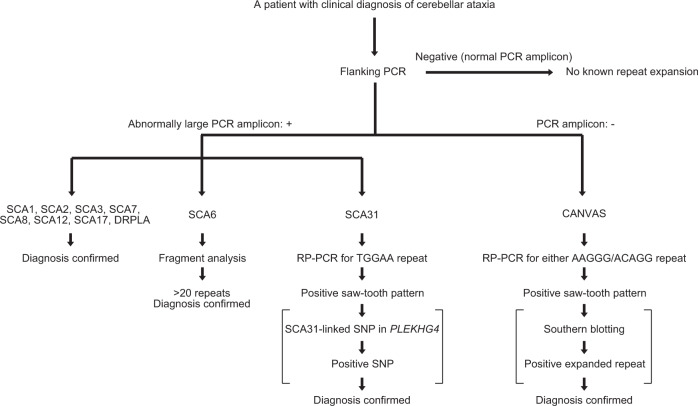
Our conventional screening workflow for known repeat expansion diseases manifesting cerebellar ataxia. Square brackets indicate procedures that can be omitted for diagnosis. From this screening workflow, we excluded SCA10, SCA37, and Friedreich ataxia because of their rarity, and SCA36 because of their regional distribution in Japan. DRPLA dentatorubral-pallidoluysian atrophy, CANVAS cerebellar ataxia, neuropathy, vestibular areflexia syndrome.

For the conventional approach, flanking PCRs targeting 11 different cerebellar ataxia-associated loci were performed (Fig. 5). The first screen was judged negative; therefore, this patient was diagnosed as having no pathogenic repeat expansion. T-LRS was called the rank #1 locus as *CACNA1A* linked to SCA6, which was compatible with the patient’s phenotype. According to the data evaluation flow, this locus was judged as disease-causing because 1) an abnormal number of repeat units was detected (21 repeat units), 2) it is known to follow autosomal-dominant inheritance, and 3) it is known to have no benign sequence expansion. To confirm this, we performed flanking PCR of the *CACNA1A* locus again, and carefully checked the size of the PCR amplicon by gel electrophoresis, separating the slightly larger allele from the normal allele of the upper limit size. Fragment analysis confirmed 21 CAG repeat units (Fig. 4a). Retrospectively, this case could be diagnosed with flanking PCR with careful examination, or with fragment analysis without prior screening by flanking PCR. However, this could only be achieved with stringent care or special expertise for repeat expansion diseases and this case showed the clear advantage of T-LRS when detecting small changes in repeat numbers that can be otherwise missed by conventional methods with low resolution.

#### Patient 17

The bidirectionally transcribed CTG·CAG repeat expansion in *ATXN8OS/ATXN8*, which leads to mRNA with an expanded CUG repeat and a polyglutamine protein^27,28^, causes SCA8. Reduced penetrance occurs in SCA8, and a possible modifier of its penetrance is repeat interruption by CCG·CGG^29^. Normal alleles usually have 15–50 repeats consisting of CTA-CTG·TAG-CAG (preceding polymorphic CTA and subsequent pathogenic CTG) while pathogenic alleles have 71–1300 repeats^30^.

A large amplicon was detected by SCA8-linked flanking PCR, indicating this patient to be SCA8. T-LRS called the rank #1 locus as *ATXN8OS/ATXN8*, although it was ambiguous for apparent pathogenicity because its expanded repeat unit number was intermediate (66 repeat units), with a relatively large portion of benign CTA repeat (19 repeat units) compared with previous data showing the number of CTA repeats to be 8–15^27^, and without CCG interruption. Altogether, this locus was excluded as disease-causing. The rank #2 locus, *AXTN7* linked to SCA7, was also rejected for pathogenicity because the repeat expansion did not exceed the manifesting threshold; therefore, this patient had no pathogenic repeat expansion. After comparing the results from the two methods, our final diagnosis was “unlikely pathogenic with intermediate SCA8 repeat expansion”. We cannot completely exclude the possibility of SCA8 with intermediate expansion although repeat length and expanded repeat unit indicated a small chance of disease manifestation. This case showed the advantage of T-LRS when both the number of repeat units and the repeat sequence are important for judging pathogenicity (Fig. 4b).

#### Patients 14 and 16

SCA31 is relatively common in Japan. A 2.5–3.8-kb-long pentanucleotide repeat expansion consisting of (TGGAA)_exp_, (TAGAA)_exp_, (TAAAA)_exp_, or (TAAAATAGAA)_exp_ in the intronic region of *BEAN1* and *TK2* is found in patients; however, only TGGAA is linked to disease^31,32^. There is also a very strongly disease-associated single-nucleotide polymorphism (SNP) in the 5′-untranslated region of *PLEKHG4*^32,33^, and positive flanking PCR and disease-associated SNPs may confirm SCA31 diagnosis.

SCA31-linked flanking PCR detected a large amplicon, and RP-PCR detected a TGGAA repeat in Patients 14 and 16. Sanger sequencing confirmed that Patient 14 was negative and Patient 16 was positive for the SCA31-linked SNP. T-LRS called *BEAN1*, linked to SCA31 as the rank #1 locus for both patients, which was judged as pathogenic because abnormal numbers of repeat units were detected (repeat length was 2756 and 2915 bp corresponding to approximately 551 and 583 repeats for Patients 14 and 16, respectively), and the TGGAA sequence (249 and 269 repeats for Patients 14 and 16, respectively) was confirmed in the consensus repeat sequence in both. The SCA31-linked SNP was also targeted; therefore, genotyping information was obtained for both patients by checking the SNP in the integrative genomics viewer without additional experimentation. T-LRS may be advantageous when conventional methods require multiple tests for diagnosis. Additionally, Patient 14 showed that SCA31-linked SNP genotyping cannot be used to exclude a diagnosis of SCA31 (Fig. 4c).

### Sequence accuracy

In nanopore sequencing, sequencing accuracy depends on the library preparation kit version used, the Guppy_basecaller version used, and its basecalling model. We used kit 109 and performed basecalling using Guppy v4.3.4, v5.0.11, or v5.1.13 with the basecalling model in high accuracy (hac) mode. According to Oxford Nanopore, raw read accuracy is approximately 95% for Guppy v4.3.4 in hac mode, and 97.8% for Guppy v.5.0.11 or later in hac mode using kit 109 (https://nanoporetech.com/accuracy). When sequences were basecalled again using Guppy v6.0.6 in sup mode, raw read accuracy increased to 98.3%. We therefore performed basecalling again with sup mode for some patients whose waterfall plots had many “other” sequences within the repeat sequence because these were possibly error sequences and might be eliminated with sup mode basecalling. When the waterfall plots of consensus sequences generated in the hac mode were compared with those from the sup mode, the sup mode improved the sequencing accuracy and decreased “other” sequences (Supplementary Fig. 4a). For the AAGGG repeat expansion in *RFC1*, which causes CANVAS, sequences in Patient 19 were basecalled again with sup mode. However, this resulted in even more “other” sequences in the consensus sequence in a strand-specific manner. Manual inspection detected that most of the “other” sequences were AAGG repeat units (Supplementary Fig. 4b). We previously experienced a similar phenomenon with nanopore sequencing of a CANVAS patient with AAGGG repeat expansion^34^, so we sequenced this patient (Patient A) using high-fidelity long-read whole-genome sequencing (HiFi LR-WGS) using the PacBio Sequel II system (Pacific Biosciences, Menlo Park, CA, USA). The AAGG repeat observed in T-LRS was mostly absent by PacBio HiFi LR-WGS. Therefore, the “other” sequences in the waterfall plot of Patient 19 are likely to be sequencing/basecalling errors (Supplementary Fig. 4c). Patient 9, another CANVAS patient with AAGGG repeat expansion in *RFC1*, also showed a “noisy” waterfall plot pattern, similar to that of Patient 19, which may also be because of sequencing errors.

As another way to evaluate sequence accuracy, we correlated the repeat lengths determined by conventional methods and T-LRS using the data from Patients 1, 3, 7, 9, 13, 15, 18, and 19. Significant correlations were observed between repeat lengths determined by conventional methods and T-LRS (*P* < 0.0001, *r*^2^: 0.9822) (Supplementary Fig. 5 and Supplementary Table 3). When validating the correlation for relatively short repeat lengths (up to 150 bp) and large repeat lengths separately, small repeats (*n* = 9 alleles) showed significant correlation (*P* < 0.0001, *r*^2^: 0.9940), while large repeats (*n* = 5 alleles) did not reach a statistically significant correlation. This is reasonable because the longer the read, the greater the chance of errors. Alternatively, it may partially result from the limited number of samples used for evaluation. To address this, we increased the number of samples by adding data from previously reported samples^34^, and reanalyzed the data (*n* = 16 alleles). This confirmed that large repeats also reached significant correlation (*P* < 0.0001, *r*^2^: 0.9436).

### Sensitivity and specificity of T-LRS as a diagnostic method

Our repeat detection workflow outputs a prioritized list of repeat loci in which the loci are displayed in order of importance (i.e., large change in the patient) by comparing them to those from our 27 unaffected controls. This list does not tell the examiner which locus is pathogenic but allows them to judge whether the respective repeat locus has pathogenic repeat expansion or not. If the examiner makes this judgment following the prioritized order from the rank #1 locus, they may easily and rapidly detect pathogenic repeat expansion because pathogenic repeat expansions are all nominated as either rank #1 or #2.

As a diagnostic tool, it is important to provide sensitivity and specificity. However, because this detection flow does not call any locus disease-causing, we could not calculate specificity or sensitivity. As shown in the prioritization list of the top 20 ranked loci for all patients in the validation study (Supplementary Table 1), disease-causing repeat expansion was found in the rank #1 locus in 83.3% (10/12) of the patients, and was found within rank #1 and #2 loci in 100% (12/12) of the patients. This can be a substitute for sensitivity in this detection workflow. No patient was detected in this study with multiple expanded repeats.

As for a substitute for specificity, we checked whether SCA loci other than “true” pathogenic repeat expansions in the six SCA patients (Patients 2, 3, 5, 9, 10, and 11) in the validation study were miscalled as pathogenic repeat expansions. In these patients, other expanded repeats were denied, except for one pathogenic expansion, prior to T-LRS. As shown in Supplementary Table 4, none of the patients had miscalled SCA repeat expansion loci, and only the true pathogenic locus was detected. Thus, the substitute of specificity was 100%.

### Time-lag sampling reduces the cost of sequencing

**Fig. 6:**
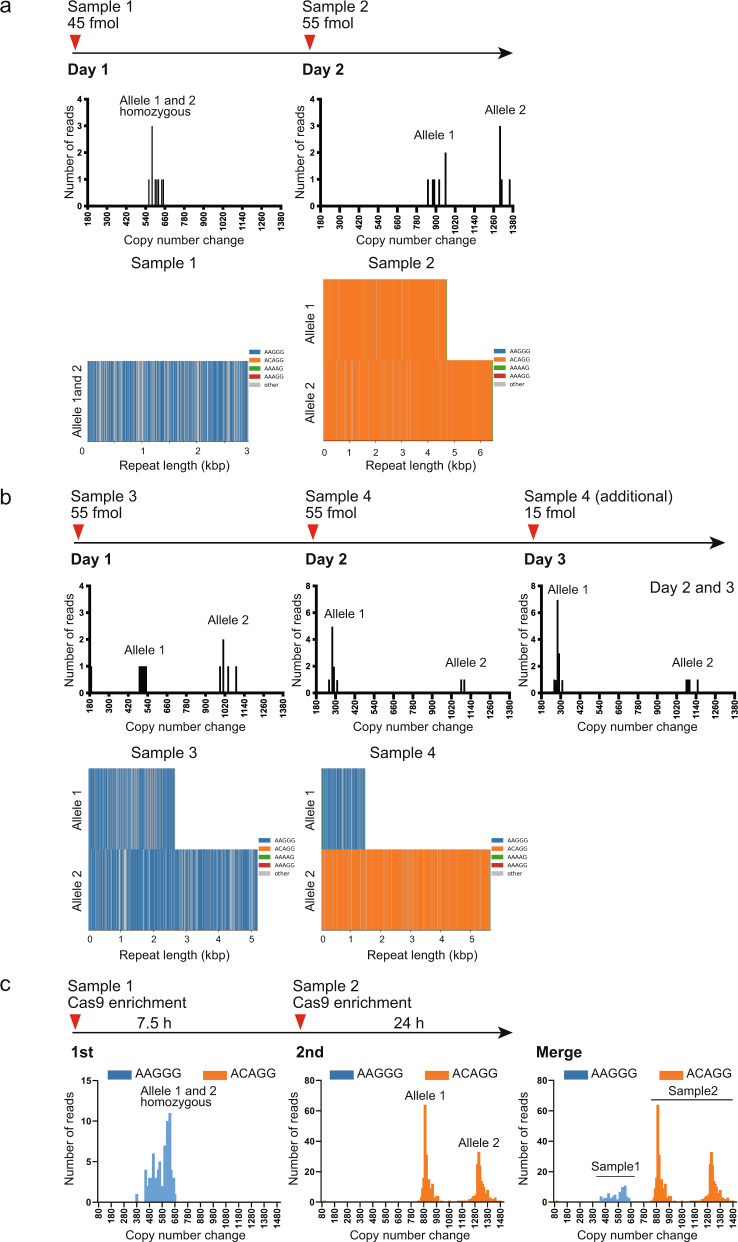
Time-lag sampling. We sequenced four patients with CANVAS, three of whom (Samples 2, 3, and 4) were previously sequenced by HiFi LR-WGS using the PacBio Sequel II system (Pacific Biosciences, Menlo Park, CA, USA)^34^. Sample 1 was identical to Patient 9. **a** Time-lag sampling of Sample 1 loading 45 fmol of library on day 1 and Sample 2 loading 55 fmol on day 2 on one GridION flow cell detected pathogenic repeat expansions in both samples. **b** Time-lag sampling of Sample 3 loading 55 fmol of library on day 1 and Sample 4 loading 55 fmol on day 2 and 15 fmol on day 3 on one GridION flow cell detected pathogenic repeat expansions in both samples. For Sample 4, all remaining library (15 fmol) was loaded again on day 3 because the sequencing output did not reach the satisfactory level at the end of day 2. **c** Time-lag sampling with Cas9-mediated PCR-free enrichment libraries of Samples 1 and 2. We sequenced Samples 1 and 2 for 7.5 and 24 h, respectively. For Sample 2, there was no AAGGG repeat expansion in its sequencing output, indicating no carryover DNA from Sample 1.

In Japan, it currently costs a minimum of 804 USD to perform one GridION run on one flow cell with two nuclease flushes and all necessary reagents. Conversely, conventional methods cost approximately 6–26 USD depending on how many experiments are needed. To reduce the sequencing cost, we tried “time-lag” sampling to sequence two different samples on one GridION flow cell using nuclease flushes (Fig. 6), which reduced the cost by approximately half (452 USD/sample). We sequenced four patients with CANVAS. Samples 2, 3, and 4 were previously sequenced by HiFi LR-WGS^34^. Sample 1 was identical to Patient 9. Two samples were serially loaded onto the same flow cell as described in Fig. 6. Mean depth of coverage for the four samples was 15.0× (11.41–16.46×). For all samples, time-lag sampling accurately detected the pathogenic repeat locus (*RFC1*). The repeat unit sequence [(AAGGG)_exp_/(AAGGG)_exp_ in samples 1 and 3, (ACAGG)_exp_/(ACAGG)_exp_ in sample 2, and (AAGGG)_exp_ /(ACAGG)_exp_ in sample 4] and the repeat length were compatible and within 10% of previous results^34^ (Supplementary Table 5). Regarding the risk of carrying over the previous library to the next sampling, Oxford Nanopore Technologies state that the wash procedure should remove 99.9% of the library, implying that some residual DNA might remain on the flow cell [Nanopore protocol Flow Cell Wash Kit (EXP-WSH004), Version: WFC_9120_v1_revB_08Dec2020]. Assuming 0.1% of the previous library is carried over, approximately 0.015× (15 × 0.001) depth in time-lag sampling might be derived from the previous library. Practically, this can be ignored.

Theoretically, the more depth the sequence output has, the greater the likelihood that the output has carried-over reads from the previous sequencing. To experimentally determine the risk of carryover in our diagnostic system, we performed time-lag sampling with Cas9-mediated PCR-free enrichment of the *RFC1* repeat locus to maximize the coverage depth using Samples 1 (repeat unit: AAGGG/AAGGG) and 2 (ACAGG/ACAGG) (Fig. 6). To maximize the depth of Sample 2, Samples 1 and 2 were sequenced for 7.5 and 24 h, respectively. The depths for Samples 1 and 2 were 163.76× and 403.02×, and there were 94 and 375 reads, respectively, which include the entire expanded repeat sequences. For Sample 2, there was no AAGGG repeat expansion in its sequencing output (Fig. 6c). Considering that our method with adaptive sampling would not have such depth, we conclude that we can use time-lag sampling with no practical concern for carrying over the previous sample.

## Discussion

Here, we developed a diagnostic method for human repeat expansion diseases using real-time T-LRS on the GridION system with the adaptive sampling option. T-LRS is a time-saving and efficient method that can simultaneously screen multiple disease-associated repeat loci of interest and obtain accurate and comprehensive data on repeat sequence and repeat length/number. T-LRS is also useful for genotyping disease-linked SNPs of interest, which can assist the diagnosis.

We showed that T-LRS has a much higher resolution than conventional methods, enabling finer detection of the repeat expansion and judgment of intermediate expansion that has a low risk of manifesting disease. This advantage improves the sensitivity and specificity of repeat disease diagnostics. Furthermore, recent CAG expansion studies in several diseases, such as SCAs or Huntington’s disease, show that intermediate, non-fully mutated repeat expansion of a locus can be associated with susceptibility to other diseases^2,35^. This can also happen with amyotrophic lateral sclerosis^36,37^, frontotemporal dementia^38^, and Alzheimer's disease^39^, although there was no such patient in our limited cohort. Therefore, high resolution with multiple locus-targeting T-LRS is preferable.

T-LRS can obtain specific pathogenic repeat unit sequence and interrupting sequence nearby. It has recently been recognized that interrupting sequence can behave as a modifier to affect genetic instability and disease course in either protective or deleterious ways in many repeat expansion disease genes, such as *FMR1*^40^, *ATXN1*^41^, *HTT*^22^ (with protective effect), and *DMPK*^25,42^ (with deleterious or protective effect). Such data would be useful to enable a patient’s disease progression/prognosis to be predicted and to inform a patient’s descendants for family planning. These data can also indicate unknown pathomechanisms or unexpected phenotype-genotype correlations.

One of the merits of our method is the ability to screen as many targets as wanted (up to 1% of the whole genome for efficient enrichment), irrespective of GC content or expanded repeat length, and output is very homogenous with uniform depth of coverage. Second, it can assign target loci without prior experimental preparation, contrary to other target-enriched systems, such as the Cas9-based approach^14^. We can therefore add a newly identified locus to a target region anytime without any experimental targeting optimization. Third, it can obtain methylation profiles in parallel. Although patients in this study were not suitable for this analysis, we showed that it could be applied to our diagnostic workflow in an ad hoc manner for patients with suspected gene silencing from hypermethylated expanded repeats and adjacent CpG islands, thereby providing additional evidence to support a diagnosis^43^.

We developed an analysis pipeline that automatically outputs the most likely disease-causing loci with prioritization after sequencing. Diagnosis may be facilitated by following the data evaluation flowchart and may be made without professional experience of repeat expansion diseases.

We also demonstrated a weak point of long-read sequencing, which is decreased raw read accuracy for specific repeat unit sequences, such as AAGGG. Regarding this problem, Tan et al. sequenced telomeres using a Nanopore sequencer with Guppy 5 and Bonito basecalling, and reported extensive basecalling-induced errors at telomere repeats. For example, up to 40–50% of (TTAGGG)_n_ repeats were miscalled as (TTAAAA)_n_, (TTAAGG)_n_, (TTAGAG)_n_, (TTGGGG)_n_, (CTTCTT)_n_ or (CCCCTGG)_n_^44^. They found that miscalling of other types of repeat occurred in a strand-specific manner and that these “other types of repeat” were not observed in the CHM13 reference genome or PacBio HiFi reads, indicating that these “other types of repeat” are artifacts of nanopore sequencing or the basecalling process, rather than biological variation^44^. The results of Tan et al. seem compatible with our sequencing results of Patient 19, who had an AAGGG repeat expansion.

An important point of this system is that it needs sufficient depth coverage to obtain reliable sequence data that avoids inaccuracies in basecalling and that can separate the two alleles for diagnosing autosomal recessive disease. To estimate the recommended coverage depth for our method, we performed down-sampling of the fastq data from Patients 10 and 18 by various proportions to set the virtual depth of the target region to 4–5×, 7–8×, 10×, 14–15×, and 20–25× (Supplementary Fig. 6). We concluded that to obtain the two separated alleles, a minimum of depth of 10–15× is needed. The amount of output data largely depends on the flow cell quality^20^; however, one way to increase the coverage depth of a single run is to shear the high-molecular-weight DNA. To detect the expansion of short TRs or minisatellites, the expected size of expansion would be up to 20 kb^2^; therefore, DNA should be sheared to approximately 40 kb.

Although T-LRS is practical and cost-saving compared with whole-genome sequencing using a long-read sequencer, it is still relatively expensive compared with conventional approaches. However, we believe the various advantages of T-LRS over conventional methods are significant. Furthermore, we propose the time-lag sampling method for reducing its cost by approximately half.

In conclusion, we propose a method for repeat expansion disease diagnostics that can meet the urgent need for rapid, accurate, and comprehensive molecular diagnosis of repeat expansion diseases.

## Methods

### Patients

Twenty-two patients with various neurological and neuromuscular diseases, including 12 with genetically diagnosed repeat expansion diseases (positive controls) and 10 with clinical diagnosis of SCA or CANVAS, but without genetic diagnosis, were analyzed. Patients 8 and 12^45^, and Patient 9^34^ were previously described. As a positive control for methylation analysis, unaffected individual 1 who had heterozygous hypermethylated repeat expansion in *NOTCH2NLC* was analyzed. This individual was recently reported as the asymptomatic father of a patient with NIID^24^, and is unrelated to Patient 6 with NIID in our cohort. The experimental protocol was approved by the Committee for Ethical Issues at Yokohama City University School of Medicine. Written informed consent was obtained from all individuals. Clinical information was collected from the medical doctors attending the patients. Genomic DNA was extracted using standard methods, from either peripheral blood leukocytes or lymphoblastoid cells established from the patient’s lymphocytes.

### Conventional repeat expansion screening

Flanking PCR, Sanger sequencing of *PHOX2B* repeat region^46^, and SCA31-linked SNP genotyping which is located in the 5′-untranslated region of *PLEKHG4* [rs886041026; *PLEKHG4* (NM_001129729.3):c.-16C>T]^33^, fragment analysis, RP-PCR, and/or southern blotting were applied for genetic diagnoses. Fragment analysis of *HTT* in Patient 1 and southern blotting of *DMPK* in Patient 4 were performed at certified clinical laboratories. Our conventional screening workflow for repeat expansion diseases that cause cerebellar ataxia is presented in Fig. 5.

### Flanking PCR

Flanking PCR surrounding repeat sequence in genic regions of *ATN1* (linked to dentatorubral-pallidoluysian atrophy)^47^, *ATXN1* (linked to SCA1)^48^, *TBP* (linked to SCA17)^49^, *PPP2R2B* (linked to SCA12)^50^, *ATXN7* (linked to SCA7)^51^, *ATXN8OS/ATXN8* (linked to SCA8)^27^, *ATXN3* (linked to SCA3)^52^, *CACNA1A* (linked to SCA6)^26^, *ATXN2* (linked to SCA2)^53^, *BEAN1* (linked to SCA31)^32^ and *RFC1* (linked to CANVAS)^54^ was performed as previously described, as the first screen of a patient with suspected SCA or/and CANVAS. Primer sequences and PCR conditions are shown in Supplementary Table 6.

### Sanger sequencing

Detailed primer sequences and PCR conditions used for Sanger sequencing of *PLEKHG4* and *PHOX2B* are shown in Supplementary Table 6. Sanger sequencing was performed using the Big Dye Terminator cycle sequencing kit (v1.1 or v3.1) (Thermo Fisher Scientific Waltham, MA, USA) on an Applied Biosystems 3500xL Genetic Analyzer (Thermo Fisher Scientific).

### Fragment analysis

Fragment analysis for *CACNA1A* was performed using the same primers and PCR settings as for flanking PCR^26^ except for the forward primer being 6-carboxyfluorescein (6-FAM)-labeled by Eurofins Genomics (Tokyo, Japan). Primer sequences and PCR conditions are shown in Supplementary Table 6.

### RP-PCR

RP-PCR for detecting GGC repeats at *NOTCH2NLC* (linked to NIID)^8^, TTTCA repeats at *SAMD12* (linked to benign adult familial myoclonic epilepsy)^10,45^, AAGGG repeats at *RFC1*^54^, and TGGAA repeats at *BEAN1* (linked to SCA31)^55^ were performed as described^8,10,45,54,55^. RP-PCR for detecting GGCCTG repeats at *NOP56* (linked to SCA36)^56^ was performed elsewhere. Primer sequences and PCR conditions are shown in Supplementary Table 6. RP-PCR products were resolved and visualized using an Applied Biosystems 3500xL Genetic Analyzer (Thermo Fischer Scientific) and analyzed using GeneMapper software (Thermo Fischer Scientific).

### Southern blotting

Patients 8 and 12, on whom southern blotting analysis was performed to detect *SAMD12* repeat expansion and *CSTB* repeat expansion, respectively, were described previously^45^. Southern blotting for *RFC1* intronic repeats was performed as previously reported^54^. In detail, 3 or 5 µg of genomic DNA was digested overnight with EcoRI-HF (New England Biolabs, Ipswich, MA, USA) to screen for *RFC1* repeat expansions. Digested DNA was separated on 0.8% agarose gels in 1.0× Tris-Borate-ethylenediaminetetraacetic acid buffer. The gel was depurinated with 0.25 M HCl for 8 min, and then denaturing DNA was done with 0.5 M NaOH/1.5 M NaCl for 15 min, 2 times. The digested DNA was transferred to positively charged nylon membranes. DNA fragments were fixed using the autocrosslinking mode of the UV Stratalinker 2400 (Stratagene, La Jolla, CA, USA). Prehybridization was performed at 37 °C for 60 min in Digoxigenin (DIG) Easy Hyb buffer (Sigma-Aldrich, St. Louis, MI, USA). DIG-labeled probes were amplified by PCR of a genomic fragment with the forward primer 5’-ATTAGGTGTCTGGTGAGGGC-3’ and the reverse primer 5’-GAAGAATGGCCCCAAAAGCA-3’ (Eurofins Genomics). The PCR products were cloned into a pCR4-TOPO Vector (Invitrogen, Waltham, MA, USA). DIG-labeled *RFC1*-PCR probe was generated using the PCR DIG Probe Synthesis Kit (Roche, Basel, Switzerland) according to the manufacturer’s instructions. Hybridization was performed at 37 °C overnight in DIG Easy Hyb buffer containing with denatured *RFC1*-PCR-labeled probe. After hybridization, membranes were washed twice at room temperature in 2× saline-sodium citrate (SSC) containing 0.1% sodium dodecyl sulfate (SDS) for 5 min, followed by two 15-min washes in 0.5× SSC containing 0.1% SDS at 65 °C. The DIG-labeled probes were detected by chemiluminescence, using anti-DIG antibodies conjugated with alkaline phosphatase (Anti-Digoxigenin-AP, Fab fragments, Roche) and its chemiluminescence substrate CDP-star (Roche). Briefly, membranes were blocked for 30 min in 1× blocking solution and then incubated for 30 min in antibody solution (75 mU/mL anti-DIG-AP), followed by two 15-min washes in washing buffer (0.1 M maleic acid, 0.15 M NaCl, 0.3% Tween 20) at room temperature. The signals were visualized on a ChemiDoc Touch (Bio-Rad, Hercules, CA, USA). The membrane was re-probed with custom-made DIG-labeled probes for AAGGG repeat detection ([DIG]-AAGGGAAGGGAAGGGAAGGGAAGGGAAGGGAAGGGAAGGGAAGGG) and ACAGG repeat detection ([DIG]-ACAGGACAGGACAGGACAGGACAGGACAGGACAGGACAGGACAGG) (probes synthesized by Eurofins Genomics).

### Real-time T-LRS using adaptive sampling on GridION

Our workflow is presented in Fig. 3. Approximately 2–3 µg of unsheared, purified genomic DNA or DNA sheared to a target size of 40 kb using a Megaruptor 2 (Diagenode, Seraing, Belgium) was used to construct sequencing libraries using the Oxford Nanopore Ligation Sequencing Kit (SQK-LSK109) (Oxford Nanopore Technologies, Oxford, UK) following the manufacturer’s instructions. As an exception to these instructions, enzyme incubation times were doubled as suggested by the manufacturer’s instruction, with the final AMPure purification incubation lasting for 10 min at 37 °C. Approximately 30–50 fmol of library was loaded onto a flow cell (FLO-MIN106D, R9.4.1) on a GridION sequencer (Oxford Nanopore Technologies). Target regions comprising 0.2% of the whole genome were enriched using the adaptive sampling option^18^ of GridION Mk1 with a bed file assigning 59 loci associated with repeat expansion diseases and each of their surrounding 100 kb regions plus the SCA31-linked SNP in *PLEKHG4* and its surrounding 40 kb region (Supplementary Table 7)^34^. For the technical validation of time-lag sampling, we performed Cas9-mediated PCR-free enrichment for targeting the *RFC1* repeat locus in accordance with the manufacturer’s protocols (Nanopore protocol for Cas9-targeted sequencing, ENR_9084_v109_revO_04Dec2018, Oxford Nanopore Technologies) and as previously described^24,45,57^. The four Alt-R® crRNAs (5′-GACAGUAACUGUACCACAAU-3′ and 5′-ACCACUAGCCAAUGCCUGUU-3′ on the plus strand; 5′-CUAUAUUCGUGGAACUAUCU-3′ and 5′-UAGGACAUUCGGAAAUUCUU-3′ on the minus strand) were mixed and used.

Sequencing was performed in hac mode for approximately 2 to 3 days with one or two additional library loading(s) after nuclease flushing of a flow cell using the Flow Cell Wash Kit (EXP-WSH004) (Oxford Nanopore Technologies). For the time-lag samplings with adaptive sampling target capture, we loaded Sample 1 to the GridION on the first day, and Sample 2 on the second and third days after the nuclease flushes. For the time-lag sampling with Cas9-mediated PCR-free enrichment, we loaded Sample 1 to the GridION on the first day and sequenced for 7.5 h. Sample 2 was loaded after the nuclease flushes and sequenced for 24 h. These procedures are described in Fig. 6.

### Data analysis

We built a data analysis pipeline using the analysis tools mentioned below, and evaluated the output data from the pipeline, following the data evaluation workflow presented in Fig. 3. Sequences were basecalled using Guppy v4.3.4, v5.0.11, or v5.1.13 in hac mode during the run on the GridION. According to Oxford Nanopore, sup mode increases the raw read accuracy; therefore, sequences were basecalled again using Guppy v6.0.6 in sup mode for Patients 4, 5, 6, 10, 11, 12, 14, and 16. They were then aligned to GRCh38 using either minimap2 v2.14 (https://github.com/lh3/minimap2) or LAST 1132 (https://gitlab.com/mcfrith/last). Depth of coverage was calculated using mosdepth v0.3.1 (https://github.com/brentp/mosdepth). Median and range of coverage depth across all 59 loci among 22 patients were calculated (Supplementary Fig. 1). Tandem-genotypes v1.3.0, v1.8.2, or v1.9.0 (https://github.com/mcfrith/tandem-genotypes) was used to find changes in the length of TRs of selected loci. Histograms of the output were drawn using tandem-genotypes-plot command, where x-axis, “copy number change”, indicates difference in the number of repeat units relative to the reference human genome, while red and blue bars indicate the forward- and the reverse-strand reads. Crude allele prediction was performed with the tandem-genotypes -o2 option, and they are shown as consensus differences in the numbers of repeat units compared with reference human genome sequence for each of the two alleles (Supplementary Tables 1 and 2). For prioritization of pathogenic repeat expansion within detected repeats, tandem-genotypes-join was performed against data from 27 controls sequenced using PromethION (Oxford Nanopore Technologies). These 27 controls were healthy Japanese individuals with no neurodegenerative disorders. For the 46 out of 59 targeted loci that are especially relevant to known repeat expansion diseases that follow Mendelian inheritance, the distribution of the number of repeat units in 54 alleles from our 27 control samples is plotted in Supplementary Fig. 7. Consensus sequences were constructed using lamassemble 1.4.2 (https://gitlab.com/mcfrith/lamassemble) or the tandem-genotypes merge option implemented in version 1.8.0 or later. When pathogenic repeat expansion was detected, we proceeded to the additional analyses (in an ad hoc manner), including detailed repeat analysis and methylation analysis. Detailed repeat analyses were performed using RepeatAnalysisTools, including the generation of waterfall-style plots from a consensus fasta file of the region of interest (waterfall plot) or coverage plots of the mapped reads (https://github.com/PacificBiosciences/apps-scripts/tree/master/RepeatAnalysisTools). When abnormal methylation was suspected, methylation analysis was performed as described previously^24^, except that we used Guppy v6.0.6 basecaller to detect 5-methylcytosine with the configuration file named “dna_r9.4.1_450bps_modbases_5mc_hac.cfg”, and we employed default settings for methylation detection and in silico bisulfite-like conversion.

To evaluate the accuracy and efficiency of target enrichment, we generated quality-filter passed fastq files, excluding reads of 1000 bp or less, which are judged by GridION to be “out of target” reads and are quit from sequencing on the adaptive sampling principle, using Filtlong v0.2.1 (https://github.com/rrwick/Filtlong). Then, we calculated the depth of coverage against both on and off-target regions among all selected reads using mosdepth. For on-targets, the average “per-locus depth of coverage” was calculated for all loci. For off-targets, bam files, including reads outside the targeted regions assigned by the bed file, were generated using samtools view -L option, and the coverage depth for every bin of 5000 bp was calculated using mosdepth. The averages of on- and off-target coverage depth data were compared to evaluate enrichment efficiency.

For down-sampling of the fastq data, we used seqkit v0.11.0 with the “sample” command by assigning the various proportions of down-sampling with -p option to set the virtual depth of the target region at 4–5×, 7–8×, 10×, 14–15× and 20–25× (https://bioinf.shenwei.me/seqkit/usage/#sample). We performed the down-sampling on fastq data from Patients 10 and 18.

### Correlation analysis between conventional methods and T-LRS on GridION

The repeat length detected by any of the conventional methods (fragment analysis, Sanger sequencing, or southern blotting) and T-LRS were compared, and the correlation between the two for Patients 1, 3, 7, 9, 13, 15, 18, and 19 was analyzed statistically by the linear regression model using GraphPad Prism v8.0.2 (GraphPad Software, San Diego, CA USA). A *P* value <0.05 was considered statistically significant.

### Reporting summary

Further information on research design is available in the Nature Research Reporting Summary linked to this article.

## Supplementary information

Reporting Summary

Supplementary material

Supplementary Table 1 (Excel spreadsheet)

Supplementary Table 2 (Excel spreadsheet)

Supplementary Table 4 (Excel spreadsheet)

Supplementary Table 6 (Excel spreadsheet)

## Data Availability

The datasets for this article are not publicly available because of concerns regarding patients’ anonymity (our data are considered as a personal identifier since it contains more than nine loci of 4-bp short tandem repeats (STRs), which is defined as a personal identifier under the Japanese law concerning protection of personal information). Requests to access the datasets from qualified researchers should be directed to the corresponding author. There are restrictions on a qualified researcher accessing the data (non-commercial use only and requiring a Data Usage Agreement).

## Code availability

All computational tools used in this study are available as open-source software, and their download links are presented in Methods. Software used are: Guppy v4.3.4, v5.0.11, v5.1.13, or v6.0.6 (https://nanoporetech.com/), minimap2 v2.14 or v2.23 (https://github.com/lh3/minimap2), LAST 1132 (https://gitlab.com/mcfrith/last), mosdepth v0.3.1 (https://github.com/brentp/mosdepth), Tandem-genotypes v1.3.0, v1.8.2, or v1.9.0 (https://github.com/mcfrith/tandem-genotypes), lamassemble 1.4.2 (https://gitlab.com/mcfrith/lamassemble), RepeatAnalysisTools (https://github.com/PacificBiosciences/apps-scripts/tree/master/RepeatAnalysisTools), methyl-stat that is a collection of command-line tools for analyzing methylation-aware oxford nanopore sequencing data including methyl-stat, methylcall, and ont2bisul (https://github.com/bitsyamagu/methyl-stat), Filtlong v0.2.1 (https://github.com/rrwick/Filtlong), seqkit v0.11.0 (https://github.com/shenwei356/seqkit), and samtools v1.3.1 or 1.9 (https://github.com/samtools/samtools). As a specific parameter, we used -r 50000 --end-bonus 10000 –no-end-fit for better mapping of long repeat regions using minimap2 as described previously^19^.
