# Supplementary material for: Rapid and comprehensive diagnostic method for repeat expansion diseases using nanopore sequencing
Source: NPJ Genom Med. 2022 Oct 26;7:62. doi: 10.1038/s41525-022-00331-y (PMC9606279; doi:10.1038/s41525-022-00331-y)
Supplement: Supplementary file 1 — Reporting Summary [file 41525_2022_331_MOESM1_ESM.pdf]

nature portfolio

Corresponding author(s): Naomichi Matsumoto

Last updated by author(s): Aug 12, 2022

Reporting Summary

Nature Portfolio wishes to improve the reproducibility of the work that we publish. This form provides structure for consistency and transparency in reporting. For further information on Nature Portfolio policies, see our Editorial Policies and the Editorial Policy Checklist.

Statistics

For all statistical analyses, confirm that the following items are present in the figure legend, table legend, main text, or Methods section.

n/a	Confirmed	
☐	☒	The exact sample size (*n*) for each experimental group/condition, given as a discrete number and unit of measurement
☐	☒	A statement on whether measurements were taken from distinct samples or whether the same sample was measured repeatedly
☒	☐	The statistical test(s) used AND whether they are one- or two-sided<br>*Only common tests should be described solely by name; describe more complex techniques in the Methods section.*
☒	☐	A description of all covariates tested
☒	☐	A description of any assumptions or corrections, such as tests of normality and adjustment for multiple comparisons
☒	☐	A full description of the statistical parameters including central tendency (e.g. means) or other basic estimates (e.g. regression coefficient) AND variation (e.g. standard deviation) or associated estimates of uncertainty (e.g. confidence intervals)
☒	☐	For null hypothesis testing, the test statistic (e.g. *F*, *t*, *r*) with confidence intervals, effect sizes, degrees of freedom and *P* value noted<br>*Give P values as exact values whenever suitable.*
☒	☐	For Bayesian analysis, information on the choice of priors and Markov chain Monte Carlo settings
☒	☐	For hierarchical and complex designs, identification of the appropriate level for tests and full reporting of outcomes
☒	☐	Estimates of effect sizes (e.g. Cohen's *d*, Pearson's *r*), indicating how they were calculated

Our web collection on statistics for biologists contains articles on many of the points above.

Software and code

Policy information about availability of computer code

Data collection	T-LRS raw data collected using Oxford Nanopore MinKNOW software (v20.10.6, v21.02.5, v21.05.8, or v21.11.7).

Data analysis

T-LRS sequences were basecalled using guppy v4.3.4, v5.0.11, or v5.1.13 with high accuracy mode and v6.0.6 for super accuracy mode. T-LRS reads were aligned to GRCh38 using either minimap2 (version 2.14 or 2.23) (https://github.com/lh3/minimap2) or LAST 1132 (https://gitlab.com/mcfrith/last). Depth of coverage was calculated using mosdepth v0.3.1 (https://github.com/brentp/mosdepth). Tandem-genotypes v1.3.0, v1.8.2 or v1.9.0 (https://github.com/mcfrith/tandem-genotypes) was used to detect the length of tandem repeats of selected loci. Histogram of the output was drawn using tandem-genotypes-plot command. Crude allele prediction was performed with the tandem-genotypes -o2 option, and they are shown as consensus differences in the numbers of repeat units compared with reference human genome sequence for each of the two alleles. Consensus sequences were constructed using lamassemble 1.4.2 (https://gitlab.com/mcfrith/lamassemble) or tandem-genotypes merge option implemented in version 1.8.0 or later. Detailed repeat analyses were performed using RepeatAnalysisTools such as generating waterfall-style plots from a consensus fasta file of the region of interest (waterfall plot) or coverage plots of the mapped reads (https://github.com/PacificBiosciences/apps-scripts/tree/master/RepeatAnalysisTools). For methylation analysis, Guppy (v6.0.6) basecaller was used to detect 5-methylcytosine, and down-stream analyses were performed using custom-tools, methyl-stat (https://github.com/bitsyamagu/methyl-stat/tree/main) with the configuration file named “dna_r9.4.1_450bps_modbases_5mc_hac.cfg”, and we employed default settings for methylation detection and in-silico bisulfite-like conversion. We used Filtlong v0.2.1 (https://github.com/rrwick/Filtlong) to generate quality-filter passed fastq files excluding reads with a length of 1000 bp or less as "on targets fastq" and samtools view -L option to generate bam files including reads outside the targeted regions assigned by the bed file as "off targets bam". We used seqkit v0.11.0 with the "sample" command for down-sampling (https://bioinf.shenwei.me/seqkit/usage/#sample), and samtools v1.3.1 or 1.9 for various analysis with bam files (http://www.htslib.org/). As a specific parameter, we used -r 50000 --end-bonus 10000 –no-end-fit for better mapping of long repeat regions using minimap2. Data visualizations were done using GraphPad Prism v8.0.2.

For manuscripts utilizing custom algorithms or software that are central to the research but not yet described in published literature, software must be made available to editors and reviewers. We strongly encourage code deposition in a community repository (e.g. GitHub). See the Nature Portfolio guidelines for submitting code & software for further information.

Data

Policy information about availability of data

All manuscripts must include a data availability statement. This statement should provide the following information, where applicable:

- Accession codes, unique identifiers, or web links for publicly available datasets
- A description of any restrictions on data availability
- For clinical datasets or third party data, please ensure that the statement adheres to our policy

Requests to access the data sets should be directed to the corresponding author, and the de-identified data are available to qualified researchers upon the reasonable request.

Human research participants

Policy information about studies involving human research participants and Sex and Gender in Research.

Reporting on sex and gender	Not relevant.
Population characteristics	Not relevant.
Recruitment	Twenty-two patients with various neurological and neuromuscular diseases, including 12 with genetically diagnosed repeat expansion diseases (positive controls) and 10 with clinical diagnosis of SCA or CANVAS, but without genetic diagnosis, were analyzed. One individual who is an unaffected carrier of certain repeat expansion was also analyzed.
Ethics oversight	The experimental protocol was approved by the Committee for Ethical Issues at Yokohama City University School of Medicine.

Note that full information on the approval of the study protocol must also be provided in the manuscript.

Field-specific reporting

Please select the one below that is the best fit for your research. If you are not sure, read the appropriate sections before making your selection.

☒ Life sciences ☐ Behavioural & social sciences ☐ Ecological, evolutionary & environmental sciences

For a reference copy of the document with all sections, see nature.com/documents/nr-reporting-summary-flat.pdf

Life sciences study design

All studies must disclose on these points even when the disclosure is negative.

Sample size	Twenty-two patients with various neurological and neuromuscular diseases plus one unaffected individual (validation cohort: n=13 , discovery cohort: n=10).
Data exclusions	No data were excluded.

Replication	Not relevant.
Randomization	Not relevant.
Blinding	Not relevant.

Reporting for specific materials, systems and methods

We require information from authors about some types of materials, experimental systems and methods used in many studies. Here, indicate whether each material, system or method listed is relevant to your study. If you are not sure if a list item applies to your research, read the appropriate section before selecting a response.

Materials & experimental systems

n/a	Involved in the study
☒	☐ Antibodies
☒	☐ Eukaryotic cell lines
☒	☐ Palaeontology and archaeology
☒	☐ Animals and other organisms
☐	☒ Clinical data
☒	☐ Dual use research of concern

Methods

n/a	Involved in the study
☒	☐ ChIP-seq
☒	☐ Flow cytometry
☒	☐ MRI-based neuroimaging

Clinical data

Policy information about clinical studies

All manuscripts should comply with the ICMJE guidelines for publication of clinical research and a completed CONSORT checklist must be included with all submissions.

Clinical trial registration	Not relevant.
Study protocol	The experimental protocol was approved by the Committee for Ethical Issues at Yokohama City University School of Medicine.
Data collection	Clinical information was collected from the medical doctors attending the patients.
Outcomes	Not relevant.
