# Supplementary material for: Rapid and comprehensive diagnostic method for repeat expansion diseases using nanopore sequencing
Source: NPJ Genom Med. 2022 Oct 26;7:62. doi: 10.1038/s41525-022-00331-y (PMC9606279; doi:10.1038/s41525-022-00331-y)
Supplement: Supplementary file 2 — Supplementary material [file 41525_2022_331_MOESM2_ESM.pdf]

Supplementary information for

Rapid and comprehensive diagnostic method for repeat expansion diseases using nanopore sequencing

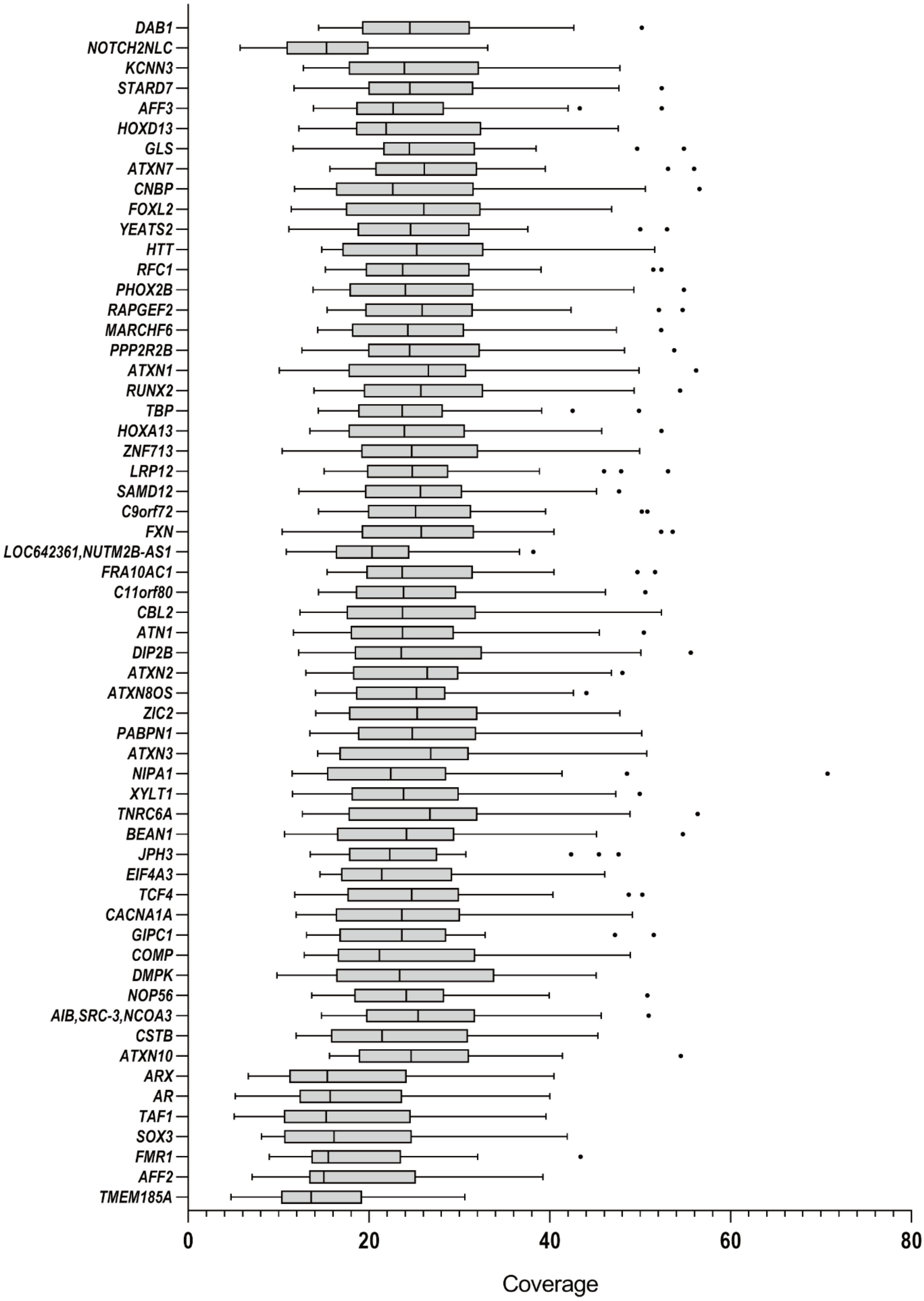

Supplementary Figure 1 Box plot showing depth of coverage across all 59 targeted loci among the 22 patients of this study

Vertical line in the box indicates median coverage and dots indicate outliers.

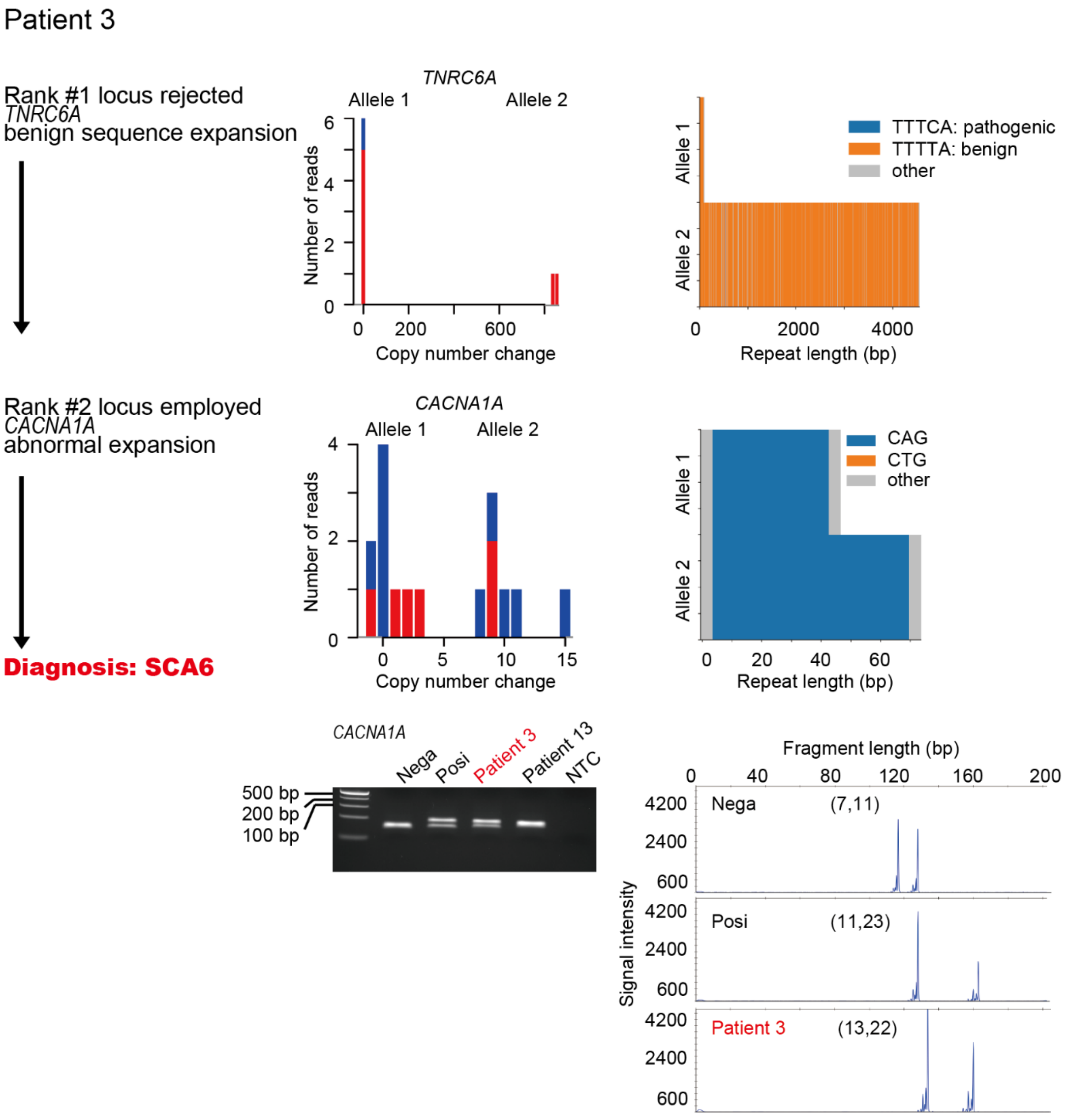

Supplementary Figure 2 An example of successful identification of pathogenic repeat expansions in Patient 3

TNRC6A repeat expansion, which was ranked #1, was judged to be a polymorphism by examining the consensus sequence constructed from our flow.

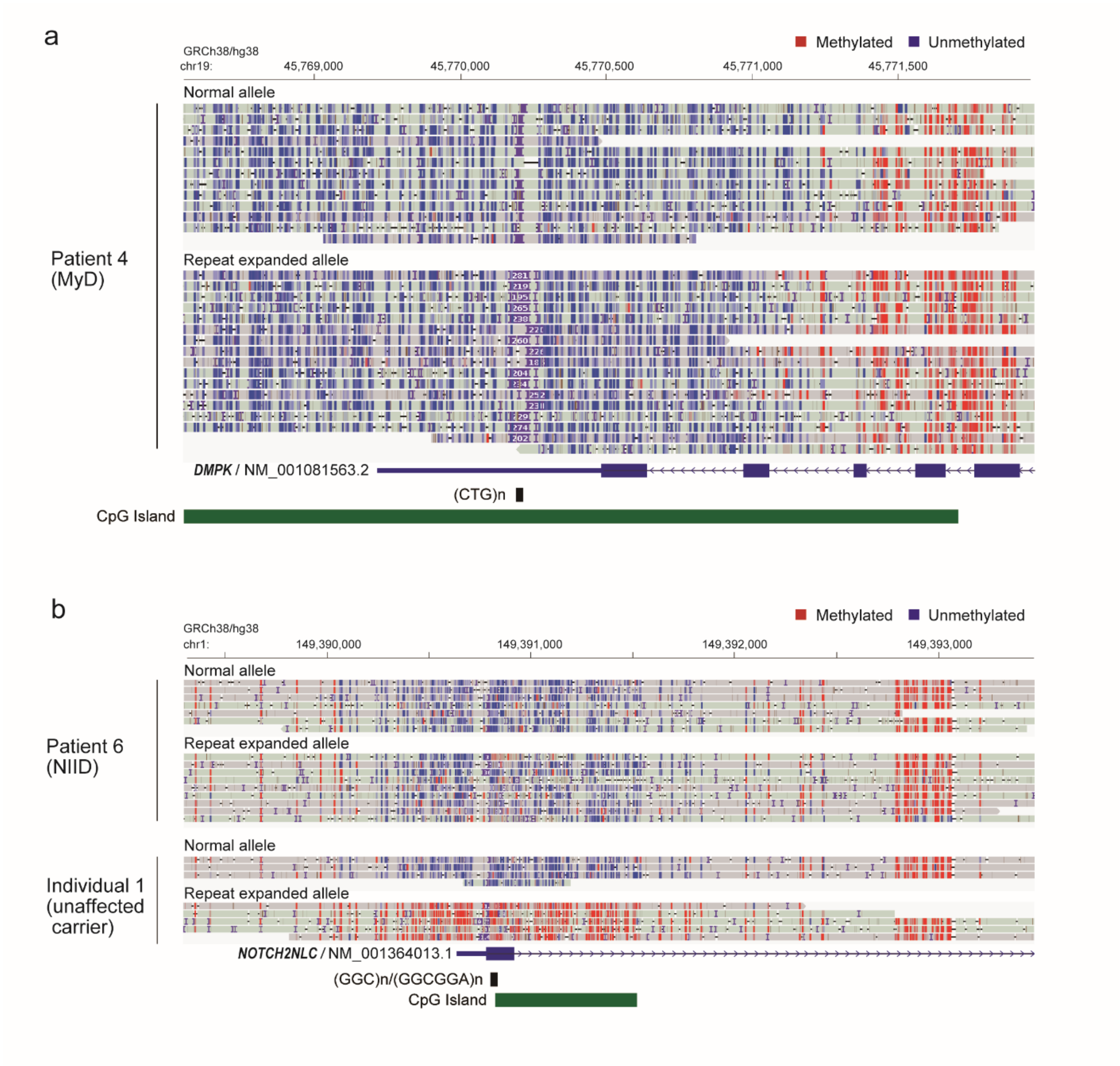

Supplementary Figure 3 Methylation analysis in Patients 4 and 6, and Individual 1

a Methylation analysis in Patient 4 with an adult-onset, mild form of myotonic dystrophy and with a relatively short repeat expansion (approximately 100 repeats). **b** Methylation analysis in Patient 6 with NIID and asymptomatic Individual 1 with an extremely long repeat expansion in *NOTCH2NLC*. Red and blue bars indicate methylated and unmethylated cytosines at CpGs, respectively. MyD: myotonic dystrophy, NIID: neuronal intranuclear inclusion disease.

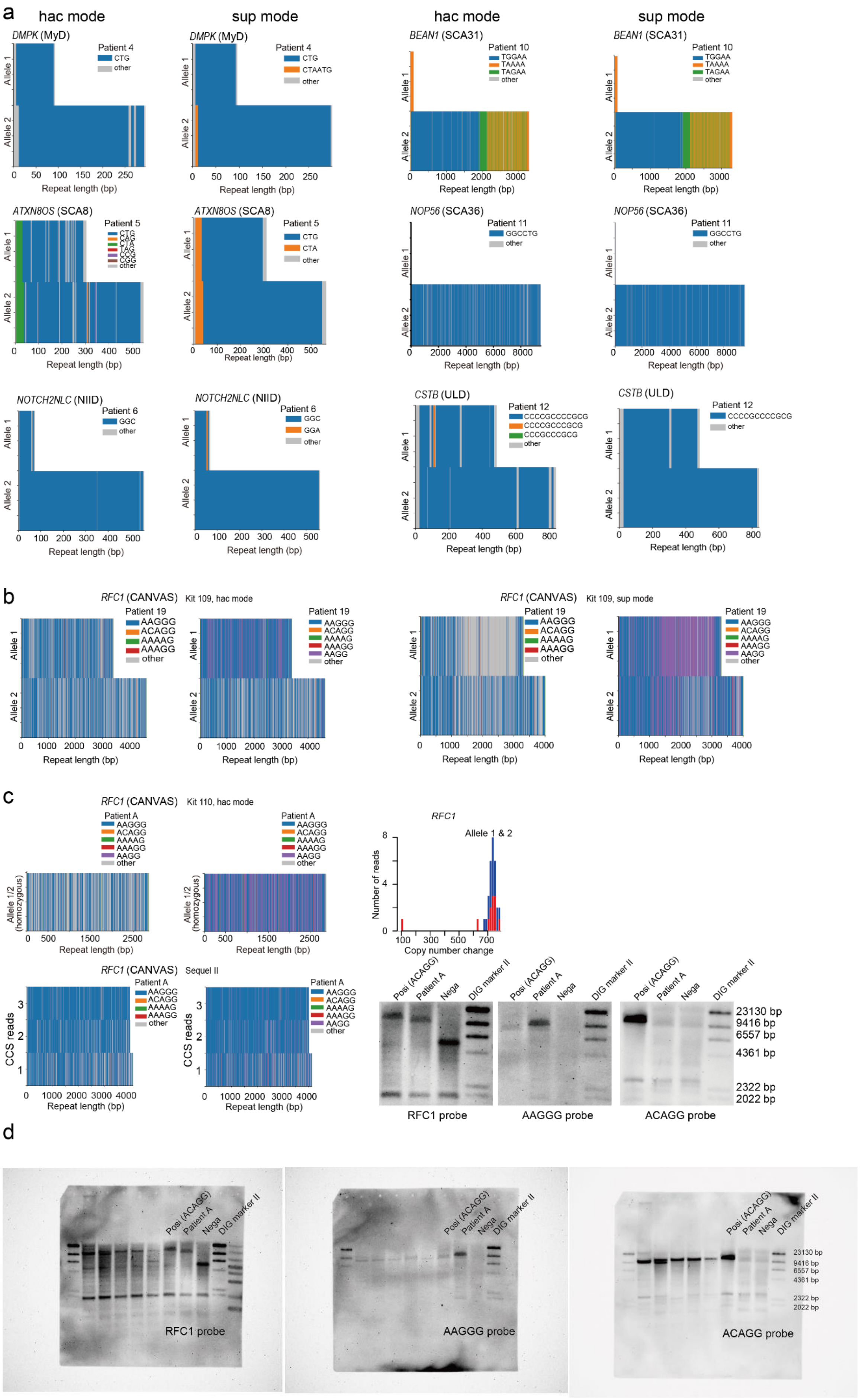

Supplementary Figure 4 Comparison of sequence accuracy between hac and sup modes

In **a** and **b**, left and right panels show consensus sequences generated under hac mode and sup mode, respectively. **a** Guppy basecalling in sup mode increased the raw read accuracy in Patients 4, 5, 6, 10, 11, and 12. Consensus sequences are depicted by waterfall plots. **b** For Patient 19 with AAGGG repeat expansion in *RFC1*, basecalling in sup mode did not increase raw read accuracy. “Other” sequences were mostly AAGG repeats. **c** Patient A with AAGGG repeat expansion in *RFC1* had been previously sequenced using T-LRS (targeted long-read sequencing) and high-fidelity long-read whole-genome sequencing (HiFi LR-WGS) using PacBio Sequel II system.[1] Upper left panel shows T-LRS sequencing using kit 110, while lower left panel shows HiFi- LR-WGS (Sequel II). In HiFi LR-WGS, the AAGG repeat was mostly absent, indicating that AAGG is the error sequence. Upper right panel shows a tandem-genotypes histogram of T-LRS showing that there was no significant strand-bias in read distribution. Lower right panel shows Southern blotting demonstrating that Patient A has AAGGG-specific repeat expansion. Note that repeat length seemed shortened in T-LRS, possibly because the repeat unit of AAGGG was recognized as AAGG. **d** Full, un-cropped images of the Southern blotting shown in **c**. The blot shown in the left panel was performed first, and then the membrane was re-probed with custom-made digoxigenin (DIG)-labeled probe for AAGGG repeat detection (shown in the middle panel) secondly and was re-probed with custom-made DIG-labeled probe for ACAGG repeat detection lastly (shown in the right panel).

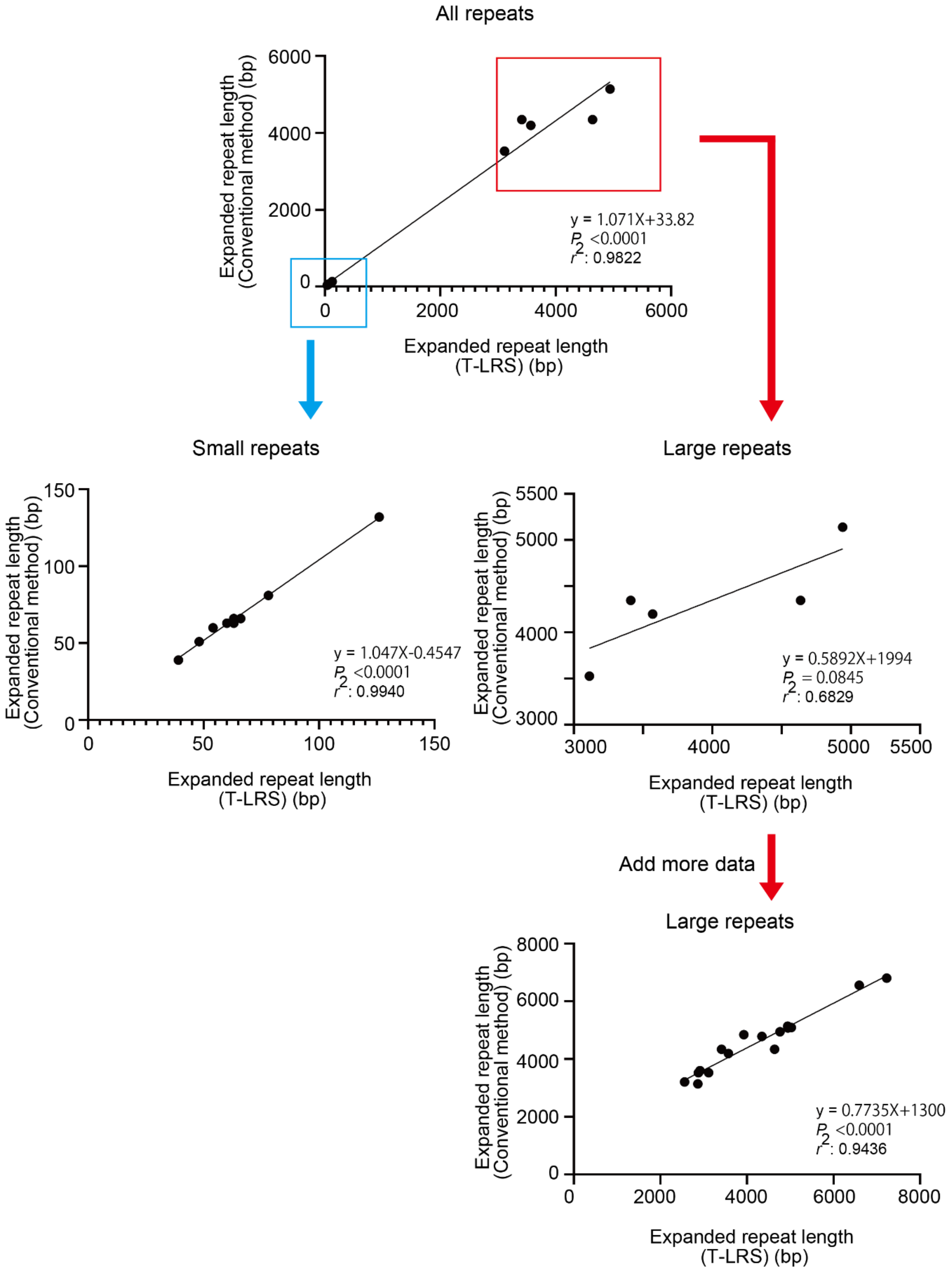

Supplementary Figure 5 Correlation analysis of repeat length between conventional methods and T-LRS

Upper panel shows overall correlation between repeat length obtained from T-LRS and conventional methods. In the middle, the left panel shows the correlation in small repeats while the right panel shows it in large repeats. Lower right panel shows significant correlation in large repeats after adding samples that were previously reported[1].

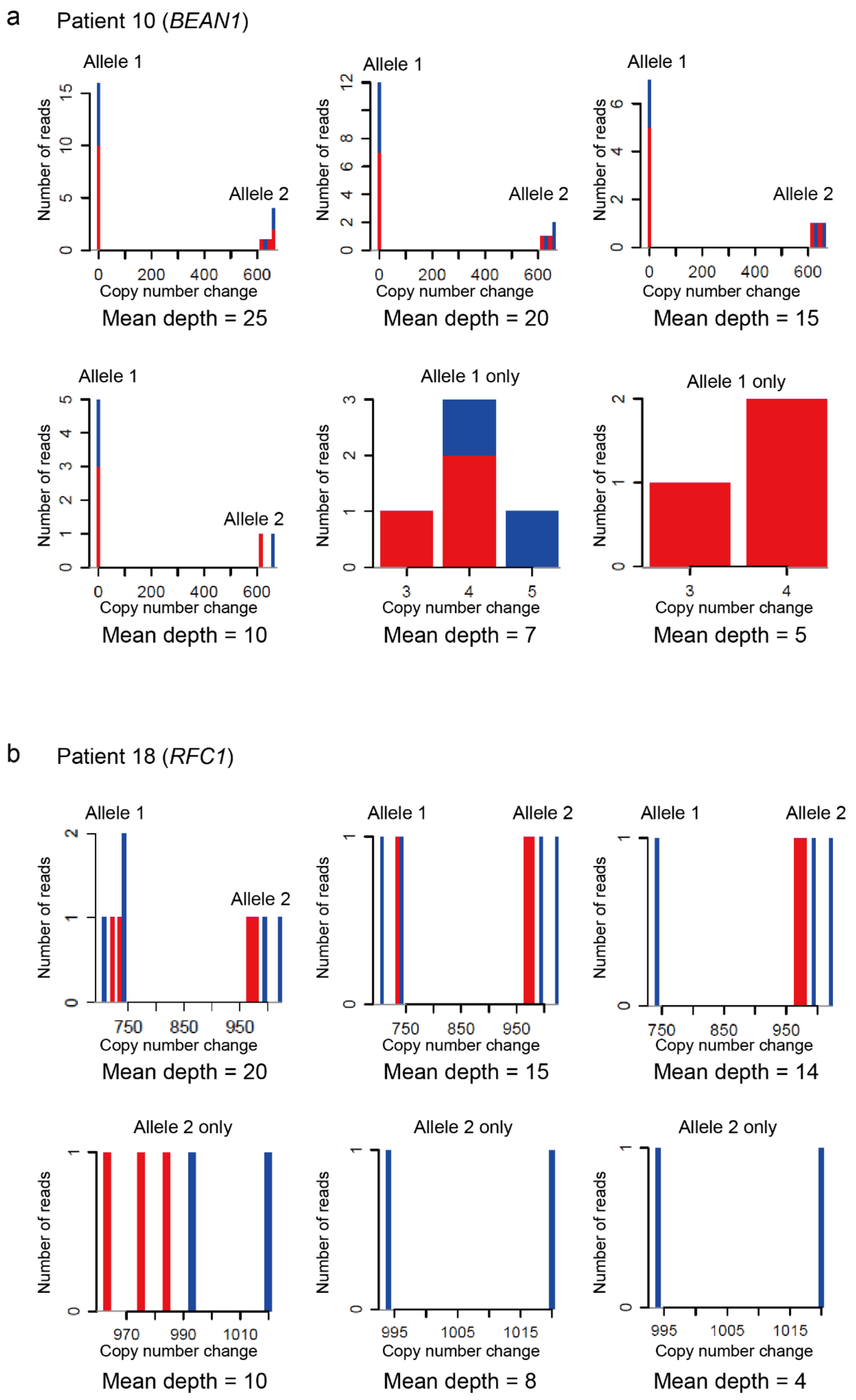

Supplementary Figure 6 Down-sampling of fastq data to estimate the minimally required depth of T-LRS to differentiate two alleles

Down-sampling at various proportions in **a** Patient 10 and **b** Patient 18. Note that with a depth of 10–15× or more, two alleles were able to be separated.

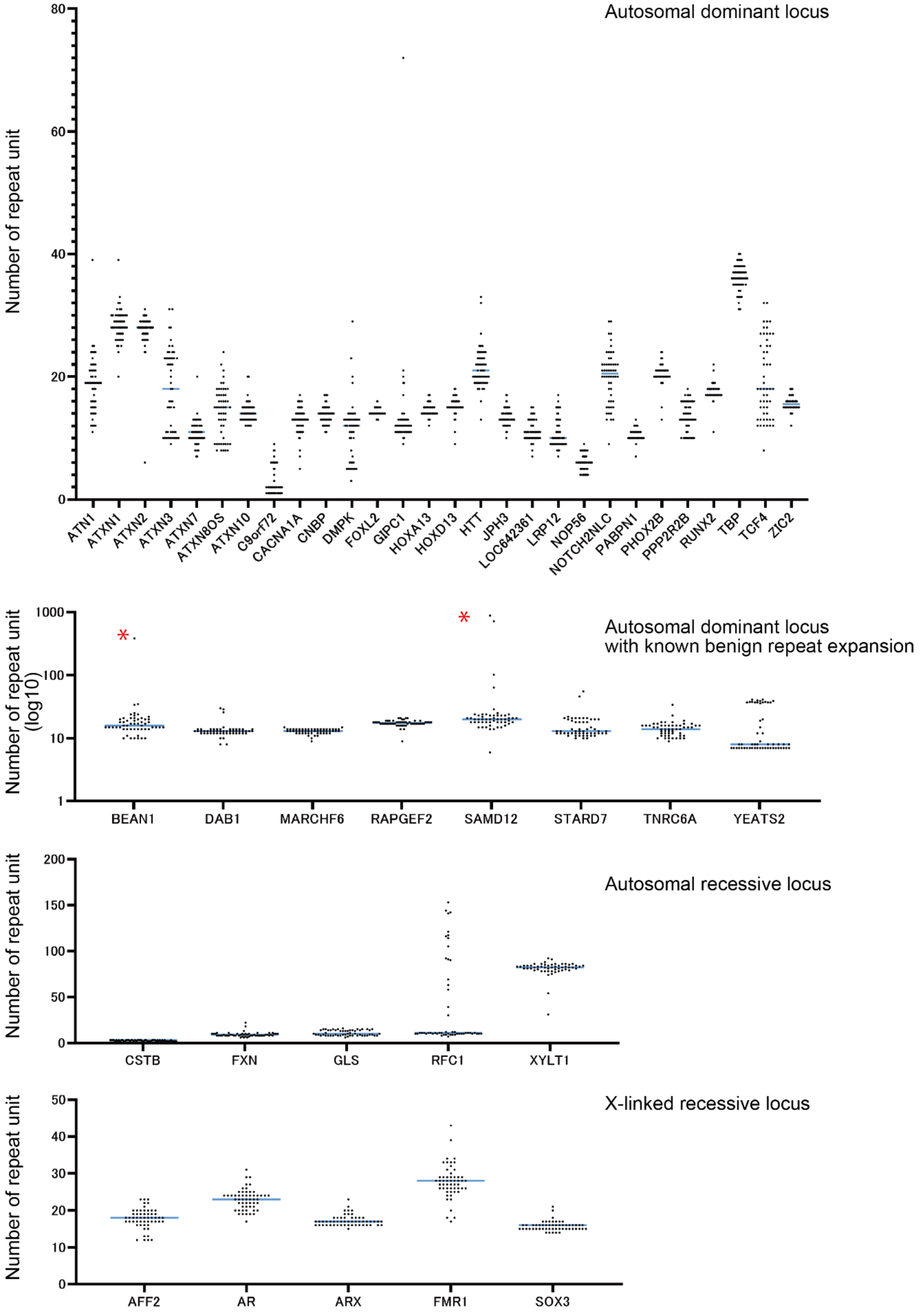

Supplementary Figure 7 Distribution of the number of repeat units in 54 alleles from our 27 control samples at 46 loci relevant to known Mendelian repeat expansion diseases

Blue horizontal bar indicates median number of repeat units. For autosomal dominant loci with known benign repeat expansion, the y-axis was set as log10 scale. There were a few expanded alleles in *BEAN1* and *SAMD12* that could not be distinguished from pathogenic expansion (*).

Supplementary Table 1 Ranked loci for possible pathogenicity in each patient in the validation study

Supplementary Table 2 Ranked loci for possible pathogenicity in each patient in the discovery study

Supplementary Table 4 Ranked loci for possible pathogenicity in six patients with SCA in the validation study and Tandem-genotypes results of SCA-associated loci for each patient

Supplementary Table 6 Primer sequences and PCR conditions used in this study

These tables are separately provided in Excel files.

Supplementary Table 3 Size comparisons of expanded repeat lengths in each patient obtained using conventional methods and T-LRS

				Repeat length (bp)		Comparison of two methods
Patient	Gene	Allele	Conventional method	Conventional method (bp)	T-LRS (bp)	Repeat length in conventional method /repeat length in T-LRS
Patient 1	*HTT*	allele 1	Fragment analysis	60	54	1.11
Patient 1	*HTT*	allele 2	Fragment analysis	132	126	1.05
Patient 3	*CACNA1A*	allele 1	Fragment analysis	39	39	1.00
Patient 3	*CACNA1A*	allele 2	Fragment analysis	66	66	1.00
Patient 7	*PHOX2B*	allele 2	Sanger sequencing	81	78	1.04
Patient 9	*RFC1*	alelle1 and 2 (homozygous)	Southern blotting	3527	3112	1.13
Patient 13	*CACNA1A*	allele 1	Fragment analysis	51	48	1.06
Patient 13	*CACNA1A*	allele 2	Fragment analysis	63	63	1.00
Patient 15	*CACNA1A*	allele 1	Fragment analysis	63	60	1.05
Patient 15	*CACNA1A*	allele 2	Fragment analysis	66	63	1.05
Patient 18	*RFC1*	allele 1	Southern blotting	4199	3569	1.18
Patient 18	*RFC1*	allele 2	Southern blotting	5140	4941	1.04
Patient 19	*RFC1*	allele 1	Southern blotting	4346	3411	1.27
Patient 19	*RFC1*	allele 2	Southern blotting	4346	4637	0.94

T-LRS: targeted long-read sequencing using adaptive sampling on GridION,

Supplementary Table 5 Sequence performance of time-lag sampling in comparison with previous data obtained from long-read sequencing

	Previous result				Time-lag sampling				Comparison of two methods	
Sample	Method	Repeat unit sequence (allele 1), (allele 2)	Repeat length (bp) (allele 1)/(allele 2)	Number of repeat units (allele 1)/(allele 2)	Mean depth (×)	Repeat unit sequence (allele 1), (allele 2)	Repeat length (bp) (allele 1)/(allele 2)	Number of repeat units (allele 1)/(allele 2)	Repeat length of allele 1 in previous result/repeat length of allele 1 in time-lag sampling	Repeat length of allele 2 in previous result/repeat length of allele 2 in time-lag sampling
Sample 1 (Patient 9)	T-LRS	(1) AAGGG, (2) AAGGG	3112	622	15.76	(1) AAGGG, (2) AAGGG	2944	589	1.06	NA
Sample 2	HiFi	(1) ACAGG, (2) ACAGG	6542/4551	1308/910	11.41	(1) ACAGG, (2) ACAGG	6478/4732	1296/946	1.01	0.96
Sample 3	HiFi	(1) AAGGG, (2) AAGGG	5154/2772	1031/554	16.46	(1) AAGGG, (2) AAGGG	5195/2669	1039/534	0.99	1.04
Sample 4	HiFi	(1) ACAGG, (2) AAGGG	5359/1552	1072/310	16.18	(1) ACAGG, (2) AAGGG	5499/1507	1100/301	0.97	1.03

Samples 2, 3, and 4 were previously sequenced[1]. HiFi: high-fidelity long-read whole-genome sequencing using PacBio Sequel II system, T-LRS: targeted long-read sequencing using adaptive sampling on GridION, Number of repeat units was calculated as the expanded repeat length divided by 5. In Sample 1 (Patient 9), homozygous AAGGG repeat expansion was detected. NA: not available due to homozygosity.

Supplementary Table 7 Targeted loci for GridION adaptive sampling

Chromosome	Start	End	Wild-type repeat sequence	Gene:disease	Site	Reference
1	149340802	149440842	GGC	*NOTCH2NLC*:NIID	5′-UTR	2
2	96147066	96247124	AAAAT	*STARD7*:FAME2	intron	2
2	176043058	176143103	GGC	*HOXD13*:SDTY5	coding	2
2	190830872	190930920	GCA	*GLS*:EIEE71	5′-UTR	2
3	63862685	63962715	GCA	*ATXN7*:SCA7	coding	2
3	129122576	129222656	CAGG	*CNBP*:DM2	intron	2
3	138896020	138996062	GCAGCT	*FOXL2*:BPES	coding	2
3	183662176	183762226	TTTTA	*YEATS2*:FAME4	intron	2
4	3024876	3124939	CAG	*HTT*:HD	coding	2
4	39237455	39416362	AAAAG	*RFC1*:CANVAS	intron	2
4	41695971	41796031	GCC	*PHOX2B*:CCHS	coding	2
4	159292526	159392618	AAAAT	*RAPGEF2*:FAME7	intron	2
5	10306338	10406411	AAAAT	*MARCHF6*:FAME3	intron	2
5	146828728	146928758	GCT	*PPP2R2B*:SCA12	intron	2
6	16277635	16377722	TGC	*ATXN1*:SCA1	coding	2
6	45372750	45472801	GGC	*RUNX2*:CCD	coding	2
6	170511907	170612021	GCA	*TBP*:SCA17	coding	2
7	27149924	27249966	GCC	*HOXA13*:HFGS	coding	2
8	104538970	104638999	CCG	*LRP12*:OPDM	5′-UTR	2
8	118316812	118416918	AAATA	*SAMD12*:FAME1	intron	2
9	27523528	27623546	GCCCCG	*C9orf72*:FTDALS1	intron	2
9	68987286	69087304	GAA	*FXN*:FRDA	intron	2
10	79776383	79876404	GGC	*LOC642361, NUTM2B-AS1*:OPDM	exon	2
12	6886716	6986773	CAG	*ATN1*:DRPLA	coding	2
12	111548950	111649019	GCT	*ATXN2*:SCA2	coding	2
13	70089383	70189428	CTG	*ATXN8OS*:SCA8	exon	2
13	99935448	100035493	GCG	*ZIC2*:HPE5	coding	2
14	23271472	23371502	GCG	*PABPN1*:OPMD	coding	2
14	92021010	92121040	CTG	*ATXN3*:SCA3	coding	2

16	24563438	24663532	AAAAT	*TNRC6A*:FAME6	intron	2
16	66440396	66540466	AATAA	*BEAN1*:SCA31	intron	2
16	87554287	87654329	GCT	*JPH3*:HDL2	intron	2
17	80096992	80197139	GCCGCTGCCGACCTCGCTGT	*EIF4A3*:RCPS	5′-UTR	2
18	55536153	55636229	AGC	*TCF4*:FECD3	intron	2
19	13157858	13257897	CAG	*CACNA1A*:SCA6	coding	2
19	14446041	14546075	CCG	*GIPC1*:OPDM	5′-UTR	2
19	45720204	45820264	CAG	*DMPK*:DM1	3′-UTR	2
20	2602733	2702757	GGGCCT	*NOP56*:SCA36	intron	2
21	43726443	43826479	CCCCGCCCCGCG	*CSTB*:ULD/EPM1	promoter	2
22	45745354	45845424	ATTCT	*ATXN10*:SCA10	intron	2
X	67495317	67595386	GCA	*AR*:SBMA	coding	2
X	24963649	25063697	GCC	*ARX*:EIEE1	coding	2
X	140454316	140554361	GGC	*SOX3*: MRGH	coding	2
X	147862050	147962110	GGC	*FMR1*:FXTAS	5′-UTR	2
X	148450637	148550682	GCC	*AFF2*:FRAXE	5′-UTR	2
1	57317044	57417080	AAAAT	*DAB1*:SCA37	intron	3
2	100055032	100155449	GCC	*AFF3*:FRA2A	5′-UTR	3
7	55837601	55937639	GCG	*ZNF713*:FRA7A	5′-UTR	3
10	93652417	93752625	CCG	*FRA10AC1*:FRA10A	5′-UTR	3
11	66694819	66794845	GGC	*C11orf80*:FRA11A	5′-UTR	3
11	119156290	119256323	CGG	*CBL2*:FRA11B	5′-UTR	3
12	50454917	50555171	GGC	*DIP2B*:FRA12A	5′-UTR	3
15	22736671	22836703	GCG	*NIPA1*:ALS	coding	3
16	17420675	17521168	GGC	*XYLT1*:BSS	promoter	3
19	18736035	18836050	CGT	*COMP*:PSACH/MED	coding	3
X	71390295	71490888	CCCTCT	*TAF1*:XDP	intron	3
X	149581570	149681808	CCG	*TMEM185A*:FRAXF	5′-UTR	4
1	154819691	154919908	GCT	*KCNN3*:Schizophrenia, migraines	3′ of gene	4
20	47601044	47701202	GCAGCA	*AIB, SRC-3, NCOA3*:Prostate, breast Cancer	coding	4
16	67260029	67300029	C>T	*PLEKHG4*:SCA31	5′-UTR	5

UTR, untranslated region. References are shown in Supplementary References. For disease, ALS: amyotrophic lateral sclerosis, BPES: blepharophimosis, ptosis and epicanthus inversus, BSS:

Baratela-Scott syndrome, CANVAS: cerebellar ataxia, neuropathy and vestibular areflexia syndrome, CCD: cleidocranial dysplasia, CCHS: congenital central hypoventilation syndrome, DM: myotonic dystrophy, DRPLA: dentatorubral-pallidoluysian atrophy, EIEE: early infantile epileptic encephalopathy, EPM: progressive myoclonus epilepsy, FAME: familial adult myoclonic epilepsy, FECD: Fuchs endothelial corneal dystrophy, FRA: fragile site, FRDA:Friedreich ataxia, FTD/ALS: frontotemporal dementia/amyotrophic lateral sclerosis, FXTAS: fragile X-associated tremor ataxia syndrome, HD: Huntington disease, HDL2:Huntington disease-like 2, HFGS: hand-foot-genital syndrome, HPE: holoprosencephaly, MED: multiple epiphyseal dysplasia, MRGH: mental retardation with isolated growth hormone deficiency, NIID: neuronal intranuclear inclusion disease, OPDM: oculopharyngodistal myopathy, OPMD: oculopharyngeal muscular dystrophy, PSAHC: Pseudoachondroplasia, RCPS: Richieri-Costa-Pereira syndrome, SBMA: spinal and bulbar muscular atrophy, SCA: spinocerebellar ataxia, SDTY: Syndactyly, ULD: Unverricht-Lundborg disease, XDP: X-linked dystonia parkinsonism.

Supplementary References

1 Miyatake, S. et al. Repeat conformation heterogeneity in cerebellar ataxia, neuropathy, vestibular areflexia syndrome. Brain **145**, 1139-1150 (2022).

2 Tang, H. *et al.* Profiling of Short-Tandem-Repeat Disease Alleles in 12,632 Human Whole Genomes. *Am. J. Hum. Genet.* **101**, 700-715 (2017).

3 Yu, J. *et al.* The GGC repeat expansion in NOTCH2NLC is associated with oculopharyngodistal myopathy type 3. *Brain* **144**, 1819-1832 (2021).

4 Castelli, L. M., Huang, W. P., Lin, Y. H., Chang, K. Y. & Hautbergue, G. M. Mechanisms of repeat-associated non-AUG translation in neurological microsatellite expansion disorders. *Biochem. Soc. Trans.* **49**, 775-792 (2021).

5 Ishikawa, K. *et al.* An autosomal dominant cerebellar ataxia linked to chromosome 16q22.1 is associated with a single-nucleotide substitution in the 5' untranslated region of the gene encoding a protein with spectrin repeat and Rho guanine-nucleotide exchange-factor domains. *Am. J. Hum. Genet.* **77**, 280-296 (2005).
